# Analogs of the Catechol Derivative Dynasore Inhibit HIV-1 Ribonuclease H, SARS-CoV-2 nsp14 Exoribonuclease, and Virus Replication

**DOI:** 10.3390/v15071539

**Published:** 2023-07-13

**Authors:** Abhishek Asthana, Angela Corona, Woo-Jin Shin, Mi-Jeong Kwak, Christina Gaughan, Enzo Tramontano, Jae U. Jung, Rainer Schobert, Babal Kant Jha, Robert H. Silverman, Bernhard Biersack

**Affiliations:** 1 Cancer Biology, Lerner Research Institute, Cleveland Clinic, 2111 East 96th St, Cleveland, OH 44106, USA; asthana@ccf.org (A.A.); shinw2@ccf.org (W.-J.S.); kwakm@ccf.org (M.-J.K.); gaughac@ccf.org (C.G.); jungj@ccf.org (J.U.J.); 2 Laboratorio di Virologia Molecolare, Dipartimento di Scienze della Vita e Dell’Ambiente, Universitá degli Studi di Cagliari, Cittadella Universitaria di Monserrato SS554, 09042 Monserrato, Italy; angela.corona@unica.it (A.C.); tramon@unica.it (E.T.); 3 Organic Chemistry 1, University of Bayreuth, Universitätsstrasse 30, 95440 Bayreuth, Germany; rainer.schobert@uni-bayreuth.de; 4 Center for Immunotherapy and Precision Immuno-Oncology, Lerner Research Institute and Department of Translational Hematology and Oncology Research, Taussig Cancer Institute, Cleveland Clinic, 2111 East 96th St, Cleveland, OH 44195, USA; jhab@ccf.org

**Keywords:** ribonucleases, enzyme inhibitors, HIV-1 RNase H, SARS-CoV-2 nsp14, coronavirus, HIV, COVID-19, antivirals

## Abstract

Viral replication often depends on RNA maturation and degradation processes catalyzed by viral ribonucleases, which are therefore candidate targets for antiviral drugs. Here, we synthesized and studied the antiviral properties of a novel nitrocatechol compound (**1c**) and other analogs that are structurally related to the catechol derivative dynasore. Interestingly, compound **1c** strongly inhibited two DEDD box viral ribonucleases, HIV-1 RNase H and SARS-CoV-2 nsp14 3′-to-5′ exoribonuclease (ExoN). While **1c** inhibited SARS-CoV-2 ExoN activity, it did not interfere with the mRNA methyltransferase activity of nsp14. In silico molecular docking placed compound **1c** in the catalytic pocket of the ExoN domain of nsp14. Finally, **1c** inhibited SARS-CoV-2 replication but had no toxicity to human lung adenocarcinoma cells. Given its simple chemical synthesis from easily available starting materials, these results suggest that **1c** might be a lead compound for the design of new antiviral compounds that target coronavirus nsp14 ExoN and other viral ribonucleases.

## 1. Introduction

Viral infections present a considerable worldwide threat to humans, as exemplified most recently by the coronavirus disease 2019 (COVID-19) pandemic of severe acute respiratory syndrome coronavirus 2 (SARS-CoV-2) that has resulted in millions of deaths [1,2,3,4,5,6]. Small molecule drugs for the treatment for SARS-CoV-2 infections were recently approved, including protease inhibitors such as nirmatrelvir (as part of the drug paxlovid) and the RNA polymerase inhibitors remdesivir and molnupiravir [7]. The efficacy of intravenous remdesivir has varied among reports, which limited its application for COVID-19 treatment [8]. Paxlovid is a relatively expensive drug expensive (USD 530 for each 5-day course) and its component ritonavir has multiple drug–drug interactions that require specific evaluation prior to usage [9]. Molnupiravir (USD 700 per 5-day course) exhibited mutagenic potential in human cells [10]. Significant progress has been made in terms of COVID-19 disease management, but currently approved small molecule therapeutics have suffered from hampered global access, limited administration routes, and poor efficacy against SARS-CoV-2 variants [11]. Although the currently circulating SARS-CoV-2 omicron variants have reduced lethality compared with prior strains, in part due to natural and vaccine-induced immunity in the population, additional antiviral drugs are being sought for new variants of concern that might escape monotherapy due to mutation acquired drug resistance [12,13].

Viral ribonucleases often play essential roles in the replication cycles of RNA viruses and retroviruses. Thus, these enzymes are suitable targets for the development of antivirals with the potential to overcome resistance to other antivirals targeting different viral enzymes. In this regard, SARS-CoV-2 has two nonstructural proteins (nsps) with ribonuclease functions, nsp14 and nsp15, which are components of the replication-transcription complex (RTC) of coronaviruses [14]. While nsp15 is an RNase A-type enzyme, the 3′-to-5′ exoribonuclease (ExoN) site of nsp14 shows similarities to RNase H enzymes, including HIV-1 RT-associated RNase H [15,16]. The ExoN domain of isolated nsp14 is inactive unless complexed with nsp10 [17]. Patulin and aurintricarboxylic acid were described as nsp14 ExoN inhibitors, which showed no synergistic effects with remdesivir [18]. In addition, ebselen was recently identified as an nsp14 ExoN inhibitor [19]. Rona et al. reported nsp14 inhibitors that had no independent direct antiviral effect against SARS-CoV-2 but synergized with remdesivir [20]. Nsp14 is a bifunctional enzyme that also harbors an mRNA capping guanine-N7-methyltransferase (MTase) activity [21]. Given that a SARS-CoV-2 ExoN knockout mutant failed to replicate, a search for novel inhibitors of this activity is justified [22].

Similarities in the active sites of HIV-1 RT-associated RNase H and SARS-CoV-2 ExoN suggest that both enzymes might be susceptible to the same antiviral drug. Both HIV-1 RNase H and SARS-CoV-2 ExoN domains are dependent on Mg^2+^ ions, which coordinate to DEDD motifs of acidic amino acids in the active sites [23,24,25]. The DEDD motif of viral proteins is a potential drug target [26]. Various catechol-based HIV-1 RNase H inhibitors, such as garcinol and a thienopyrimidine compound, were previously identified, which can coordinate Mg^2+^ of the active site, thus blocking binding and cleavage of the RNA substrate [27,28]. Dynasore, viz., (*E*)-*N*′-(3,4-dihydroxybenzylidene)-3-hydroxy-2-naphthohydrazide, is a catechol derivative that inhibits endocytosis due to interference with dynamin [29,30]. As a result, dynasore inhibited the entry of different viruses, including dengue virus, herpes simplex virus-1 and -2, Newcastle disease virus, human papillomavirus, Chikungunya virus, swine fever virus, and porcine deltacoronavirus [31,32,33,34,35,36,37]. Also, dynasore induced NF-κB and IFN-β pathways by activation of mitochondrial antiviral signaling protein (MAVS) based on ROS (reactive oxygen species) formation and Rac1 upregulation [38]. In this instance, however, dynasore reactivated latent HIV-1 by activation of miniMAVS isoforms upon induction of antioxidative stress response [39]. A notable property of dynasore is that its chemical synthesis is relatively simple, starting from commercially available compounds, which also enables the preparation of a large series of analogs for biological testing and activity optimization. Here, we identified a new dynasore derivative, active against both HIV-1 RNase H and SARS-CoV-2 ExoN, that inhibits the replication of SARS-CoV-2.

## 2. Materials and Methods

### 2.1. Chemistry 

#### 2.1.1. General Procedures and Materials

Melting points are uncorrected; IR spectra were recorded on an FT-IR spectrophotometer with ATR sampling unit; NMR spectra were run on 300 MHz spectrometers; chemical shifts are given in ppm (δ) downfield from tetramethylsilane as internal standard; mass spectra: direct inlet, EI, 70 eV; HRMS: UPLC/Orbitrap MS system in ESI mode. Starting compounds and reagents were purchased from Merck (Darmstadt, Germany), Alfa Aesar (Karlsruhe, Germany), and TCI (Zwijndrecht, Belgium). 3-Nitrovanillin and 3,4-dihydroxy-5-nitrobenzaldehyde were prepared from vanillin according to a procedure in the literature [40]. 2,3-Dihydroxy-4-methoxybenzaldehyde was prepared from the reaction of 2,3,4-trimethoxybenzaldehyde with BCl_3_, 4-(*N*-methylpiperazinyl)-benzaldehyde from treatment of 4-fluorobenzaldehyde with *N*-methylpiperazine [41,42]. Synthesis and analysis of dynasore and the known compounds **1d** and **1f**–**k** were described before [43,44,45,46,47]. All tested compounds were >95% pure by UPLC/HRMS.

#### 2.1.2. (*E*)-*N*’-(4-Hydroxy-3-nitrobenzylidene)-3-hydroxy-2-naphthohydrazide (**1a**)

3-Hydroxy-2-naphthoic hydrazide (202 mg, 1.0 mmol) and 4-hydroxy-3-nitrobenzaldehyde (167 mg, 1.0 mmol) were dissolved in EtOH (25 mL). The reaction mixture was stirred under reflux for 2 h. The formed precipitate was collected, washed with EtOH, and dried in vacuum. Yield: 305 mg (0.87 mmol, 87%); yellow solid of m.p. 276–277 °C; *υ*_max_(ATR)/cm^−1^ 3243, 3056, 1662, 1641, 1626, 1593, 1562, 1548, 1531, 1515, 1490, 1468, 1449, 1423, 1395, 1359, 1319, 1288, 1249, 1216, 1175, 1103, 1070, 1019, 949, 934, 915, 891, 882, 866, 856, 836, 811, 791, 763, 743, 705, 664; ^1^H NMR (300 MHz, DMSO-d_6_) *δ* 7.24 (1 H, d, J = 8.7 Hz), 7.3–7.4 (2 H, m), 7.5–7.6 (1 H, m), 7.7–7.8 (1 H, m), 7.9–8.0 (2 H, m), 8.24 (1 H, s), 11.2–11.5 (2 H, m), 12.00 (1 H, s); ^13^C NMR (75.5 MHz, DMSO-d_6_) *δ* 110.5, 119.7, 120.4, 123.8, 124.2, 125.7, 125.8, 126.8, 128.2, 128.6, 130.3, 133.0, 135.8, 137.1, 146.4, 153.5, 153.9, 163.6; *m/z* (%) 351 (38) [M^+^], 171 (97), 142 (28), 115 (100); HRMS for C_18_H_14_O_5_N_3_ [M^+^ + H] calcd. 352.09280, found 352.09254, for C_18_H_12_O_5_N_3_ [M^−^ − H] calcd. 350.07715, found 350.07754.

#### 2.1.3. (*E*)-*N*′-(4-Hydroxy-5-methoxy-3-nitrobenzylidene)-3-hydroxy-2-naphthohydrazide (**1b**)

Analogously to the synthesis of **1a**, compound **1b** was obtained from 3-hydroxy-2-naphthoic hydrazide (202 mg, 1.0 mmol) and 4-hydroxy-5-methoxy-3-nitrobenzaldehyde (197 mg, 1.0 mmol). Yield: 325 mg (0.85 mmol, 85%); yellow solid of m.p. 258–259 °C; *R*_f_ = 0.80 (ethyl acetate); *υ*_max_(ATR)/cm^−1^ 3195, 3160, 3065, 3004, 1644, 1620, 1598, 1558, 1540, 1513, 1477, 1450, 1416, 1385, 1361, 1322, 1281, 1226, 1205, 1153, 1074, 1065, 1017, 997, 971, 955, 920, 870, 841,816, 792, 764, 749, 736, 713; ^1^H NMR (300 MHz, DMSO-d_6_) *δ* 3.97 (3 H, s), 7.4–7.5 (2 H, m), 7.5–7.6 (1 H, m), 7.63 (1 H, s), 7.7–7.9 (2 H, m), 7.92 (1 H, d, J = 8.1 Hz), 8.42 (2 H, d, J = 7.9 Hz), 10.9–11.0 (1 H, m), 11.26 (1 H, s), 12.02 (1 H, s); ^13^C NMR (75.5 MHz, DMSO-d_6_) *δ* 56.7, 110.5, 112.2, 116.3, 120.5, 123.8, 124.9, 125.8, 126.8, 128.2, 128.6, 130.3, 135.8, 137.1, 144.3, 146.7, 149.9, 153.9, 163.6; *m/z* (%) 381 (54) [M^+^], 171 (99), 142 (37), 115 (100); HRMS for C_19_H_16_O_6_N_3_ [M^+^ + H] calcd. 382.10336, found 382.10320, for C_19_H_14_O_6_N_3_ [M^−^ − H] calcd. 380.08771, found 380.08801.

#### 2.1.4. (*E*)-*N*′-(3,4-Dihydroxy-5-nitrobenzylidene)-3-hydroxy-2-naphthohydrazide (**1c**)

Analogously to the synthesis of **1a**, compound **1c** was obtained from 3-hydroxy-2-naphthoic hydrazide (202 mg, 1.0 mmol) and 3,4-dihydroxy-5-nitrobenzaldehyde (183 mg, 1.0 mmol). Yield: 320 mg (0.87 mmol, 87%); yellow solid of m.p. 276 °C; *R*_f_ = 0.78 (ethyl acetate); *υ*_max_(ATR)/cm^−1^ 3341, 3059, 1652, 1625, 1584, 1535, 1466, 1441, 1402, 1356, 1309, 1287, 1244, 1224, 1174, 1150, 1130, 1111, 1023, 950, 919, 883, 868, 850, 836, 812, 787, 778, 757, 744, 735, 658; ^1^H NMR (300 MHz, DMSO-d_6_) *δ* 7.3–7.4 (2 H, m), 7.5–7.6 (1 H, m), 7.62 (1 H, s), 7.67 (1 H, s), 7.76 (1 H, d, J = 8.2 Hz), 7.8–7.9 (1 H, m), 8.34 (1 H, s), 8.45 (1 H, s), 10.5–10.8 (2 H, m), 11.2–11.3 (1 H, m), 11.98 (1 H, s); ^13^C NMR (75.5 MHz, DMSO-d_6_) *δ* 110.6, 115.4, 120.3, 123.8, 124.9, 125.8, 126.8, 128.2, 128.6, 130.3, 135.8, 137.2, 143.8, 146.7, 148.1, 154.0, 163.6; *m/z* (%) 367 (47) [M^+^], 171 (100), 142 (26), 115 (75); HRMS for C_18_H_14_O_6_N_3_ [M^+^ + H] calcd. 368.08771, found 368.08765, for C_18_H_12_O_6_N_3_ [M^−^ − H] calcd. 366.07206, found 366.07230.

#### 2.1.5. (*E*)-*N*’-(2,3-Dihydroxy-4-methoxybenzylidene)-3-hydroxy-2-naphthohydrazide (**1e**)

Analogously to the synthesis of **1a**, compound **1e** was obtained from 3-hydroxy-2-naphthoic hydrazide (202 mg, 1.0 mmol) and 2,3-dihydroxy-4-methoxybenzaldehyde (168 mg, 1.0 mmol). Yield: 272 mg (0.77 mmol, 77%); colorless solid of m.p. 258–259 °C; *υ*_max_(ATR)/cm^−1^ 3529, 3462, 3268, 3083, 3057, 2940, 2846, 1635, 1621, 1607, 1551, 1507, 1474, 1445, 1397, 1356, 1320, 1294, 1275, 1244, 1218, 1170, 1148, 1118, 1099, 1073, 1033, 1018, 960, 955, 935, 925, 912, 878, 843, 808, 794, 783, 767, 733, 702, 665; ^1^H NMR (300 MHz, DMSO-d_6_) *δ* 3.83 (3 H, s), 6.63 (1 H, d, J = 8.8 Hz), 7.00 (1 H, d, J = 8.8 Hz), 7.3–7.4 (2 H, m), 7.5–7.6 (1 H, m), 7.77 (1 H, d, J = 8.0 Hz), 7.92 (1 H, d, J = 7.9 Hz), 8.46 (1 H, s), 8.57 (2 H, s), 11.18 (1 H, s), 11.2–11.4 (1 H, br s), 12.0–12.1 (1 H, br s); ^13^C NMR (75.5 MHz, DMSO-d_6_) *δ* 55.8, 103.8, 110.6, 112.6, 119.9, 120.4, 123.8, 125.8, 126.7, 128.2, 128.6, 130.2, 133.9, 135.9, 146.8, 150.0, 154.1, 163.6; *m/z* (%) 352 (43) [M^+^], 171 (100), 115 (48); HRMS for C_19_H_17_O_5_N_2_ [M^+^ + H] calcd. 353.11320, found 353.11276.

#### 2.1.6. (*E*)-*N*’-[4-(*N*-Methylpiperazinyl)benzylidene]-3-hydroxy-2-naphthohydrazide (**1l**)

Analogously to the synthesis of **1a**, compound **1l** was obtained from 3-hydroxy-2-naphthoic hydrazide (202 mg, 1.0 mmol) and 4-(*N*-methylpiperazinyl)-benzaldehyde (204 mg, 1.0 mmol). Yield: 190 mg (0.49 mmol, 49%); yellow solid of m.p. 256–257 °C; *υ*_max_(ATR)/cm^−1^ 3236, 3050, 2945, 2846, 2809, 1659, 1626, 1600, 1553, 1512, 1456, 1427, 1393, 1339, 1301, 1289, 1228, 1191, 1154, 1120, 1067, 1000, 964, 951, 931, 918, 838, 816, 773, 744, 677, 657; ^1^H NMR (300 MHz, DMSO-d_6_) *δ* 2.23 (3 H, s), 2.4–2.6 (4 H, m), 3.2–3.3 (4 H, m), 7.01 (2 H, d, J = 8.9 Hz), 7.3–7.4 (2 H, m), 7.5–7.6 (1 H, m), 7.50 (2 H, d, J = 8.9 Hz), 7.75 (1 H, d, J = 8.4 Hz), 7.90 (1 H, d, J = 8.1 Hz); ^13^C NMR (75.5 MHz, DMSO-d_6_) *δ* 45.7, 47.0, 54.4, 110.6, 114.5, 120.0, 123.7, 125.8, 126.7, 128.1, 128.5, 128.6, 129.9, 135.8, 149.0, 152.2, 154.6, 163.6; *m/z* (%) 388 (100) [M^+^], 218 (47), 201 (41), 171 (43), 115 (24), 70 (28), 43 (30); HRMS for C_23_H_25_O_2_N_4_ [M^+^ + H] calcd. 389.19720, found 389.19689, for C_23_H_23_O_2_N_4_ [M^−^ − H] calcd. 387.18155, found 387.18193.

#### 2.1.7. (*E*)-*N*′-(3,4-Dihydroxy-5-nitrobenzylidene)-2-hydroxybenzohydrazide (**2a**)

Analogously to the synthesis of **1a**, compound **2a** was obtained from 2-hydroxybenzoic hydrazide (152 mg, 1.0 mmol) and 3,4-dihydroxy-5-nitrobenzaldehyde (183 mg, 1.0 mmol). Yield: 220 mg (0.69 mmol, 69%); orange solid of m.p. 275 °C (dec.); *R*_f_ = 0.77 (ethyl acetate); *υ*_max_(ATR)/cm^−1^ 3332, 3088, 1637, 1627, 1591, 1558, 1538, 1489, 1469, 1446, 1409, 1358, 1310, 1285, 1251, 1223, 1173, 1153, 1121, 1082, 1036, 1026, 953, 940, 920, 903, 866, 851, 823, 801, 791, 772, 758, 749, 696, 668; ^1^H NMR (300 MHz, DMSO-d_6_) *δ* 6.9–7.0 (2 H, m), 7.4–7.5 (1 H, m), 7.58 (1 H, s), 7.65 (1 H, s), 7.88 (1 H, d, J = 8.0 Hz), 8.33 (1 H, s), 10.67 (12 H, s), 11.86 (2 H, s); ^13^C NMR (75.5 MHz, DMSO-d_6_) *δ* 113.6, 114.5, 117.0, 117.7, 117.8, 125.0, 127.1, 133.4, 133.7, 145.4, 146.2, 147.6, 160.0, 165.4; HRMS for C_14_H_12_O_6_N_3_ [M^+^ + H] calcd. 318.07206, found 318.07169.

#### 2.1.8. (*E*)-*N*′-(3,4-Dihydroxy-5-nitrobenzylidene)-3-fluorobenzohydrazide (**2b**)

Analogously to the synthesis of **1a**, compound **2b** was obtained from 3-fluorobenzoic hydrazide (154 mg, 1.0 mmol) and 3,4-dihydroxy-5-nitrobenzaldehyde (183 mg, 1.0 mmol). Yield: 205 mg (0.64 mmol, 64%); orange solid of m.p. 251–252 °C (dec.); *R*_f_ = 0.76 (ethyl acetate); *υ*_max_(ATR)/cm^−1^ 3279, 3084, 1644, 1624, 1610, 1588, 1552, 1539, 1485, 1474, 1446, 1399, 1335, 1317, 1284, 1271, 1241, 1186, 1126, 1063, 1025, 1004, 967, 947, 940, 908, 893, 853, 830, 812, 801, 791, 762, 747, 701, 6757.34–7.8 (6 H, m), 8.32 (1 H, s), 10.70 (2 H, s), 11.93 (1 H, s); ^13^C NMR (75.5 MHz, DMSO-d_6_) *δ* 114.2–114.6 (m), 115.2–115.4 (m), 118.5–118.8 (m), 123.8, 125.0, 130.6–130.7 (m), 135.7, 137.3, 143.7, 146.4, 148.1, 160.3–163.5 (m), 161.7; HRMS for C_14_H_11_O_6_N_3_F [M^+^ + H] calcd. 320.06773, found 320.06717. 

#### 2.1.9. (*E*)-*N*′-(3,4-Dihydroxy-5-nitrobenzylidene)-nicotinoylhydrazide (**2c**)

Analogously to the synthesis of **1a**, compound **2c** was obtained from nicotinoyl hydrazide (137 mg, 1.0 mmol) and 3,4-dihydroxy-5-nitrobenzaldehyde (183 mg, 1.0 mmol). Yield: 260 mg (0.86 mmol, 86%); orange solid of m.p. 250–251 °C (dec.); *R*_f_ = 0.30 (ethyl acetate); *υ*_max_(ATR)/cm^−1^ 3310, 3256, 3065, 2603, 1662, 1626, 1594, 1538, 1475, 1425, 1297, 1279, 1252, 1146, 1121, 1075, 1032, 1021, 942, 900, 854, 828, 789, 763, 697; ^1^H NMR (300 MHz, DMSO-d_6_) *δ* 7.5–7.7 (3 H, m), 8.2–8.4 (2 H, m), 8.76 (1 H, s), 9.06 (1 H, s), 10.5–10.7 (2 H, m), 12.04 (1 H, s); ^13^C NMR (75.5 MHz, DMSO-d_6_) *δ* 115.3, 123.6, 124.9, 129.1, 135.4, 137.3, 143.8, 146.6, 148.1, 148.5, 152.2, 161.6; HRMS for C_13_H_11_O_5_N_4_ [M^+^ + H] calcd. 303.07240, found 318.07190.

### 2.2. Plasmid Construction and Protein Purification 

The genes of full-length nsp10 and nsp14 ExoN domain [1–289 amino acid (aa)] of SARS-CoV-2 (GenBank:NC_045512.2) were codon-optimized for *E. coli* expression and chemically synthesized (GenScript, Piscataway, NJ, USA). The synthesized nsp10 and nsp14 were fused to the N-terminal GST tag containing 3C protease cleavage site and the N-terminal 10His-SUMO containing TEV cleavage site, respectively. For nsp14 (ExoN)/nsp10 complex purification, two proteins were co-expressed in *E. coli* BL21(DE3) in 6 L of LB media supplemented with 50 µg/mL ampicillin and 25 µg/mL kanamycin, or 10H-SUMO-nsp14 (1–289aa) was singly expressed in *E. coli* BL21(DE3) in 6 L of LB media supplemented with 25 µg/mL kanamycin. Cultures were grown at 18 °C overnight after 0.5 mM IPTG induction. Cell pellets were resuspended using lysis buffer (250 mM NaCl and 20 mM HEPES pH 7.5) and disrupted using a microfluidizer (LM20, Microfluidics, Westwood, MA, USA). Lysates were cleared by centrifugation at 18,000 rpm, 4 °C for 60 min (Sorvall Lynx 4000) and cleared lysates were filtered using 0.45 µm membrane filter and incubated with glutathione resin (ThermoFisher, Rockford, IL, USA) for 2 h at 4 °C. The resin mixtures were loaded onto a gravity column and washed with lysis buffer, followed by elution with lysis buffer containing 10 mM glutathione. The fused tags were cleaved by TEV and 3C proteases during dialyzing in lysis buffer at 4 °C overnight, and the cleaved tags were then removed by a second round of glutathione resin purification, followed by a Histrap column (Cytiva, Marlborough, MA, USA) purification using ÄKTA Pure instrument. The fractions of nsp14-nsp10 complex were collected and further purified using a HiLoad 16/600 superdex 200 pg column (Cytiva, Marlborough, MA, USA) with lysis buffer.

For MTase activity assay full length SARS-CoV-2 nsp14 (GenBank: OU351888.1) was used. Nsp14 was PCR amplified using forward primer 5′-TTCAAGGATCCATGGCTGAAAATGTAACAGGACTC-3′ (with BamHI restriction site underlined) and reverse primer 5′-TTCAAGTCGACCTACTGAAGTCTTGTAAAAGTGTTCC-3′ (with SalI restriction site underlined). SARS-CoV-2 Nsp14 cDNA template was a gift from Prof. Wang Pei-Hui (Shandong University, Jinan, China). The amplified DNA product was cloned into cloned pGEX-6P1 vector at BamHI and SalI site and expressed as a GST fusion protein in *E. coli* strain BL21 (DE3)/ pLysS (Life Technologies, Carlsbad, CA, USA). Nsp14 purification was performed by a purification method we previously described for AKAP7 and PDE12 proteins [48].

### 2.3. HIV-1 RNase H Inhibition 

HIV-1 RT group M subtype B was expressed and purified as described [49]. The HIV-1 RT-associated RNase H inhibition assay was performed as described previously [49,50]. Briefly, anti-RNase H activity was measured in 100 µL reaction volume containing 50 mM Tris-HCl buffer pH 7.8, 6 mM MgCl_2_, 1 mM dithiothreitol (DTT), 80 mM KCl, and HIV-1 RT. Reactions were started by addition of hybrid RNA/DNA 5′-GAUCUGAGCCUGGGAGCU-fluorescein-3′ (high-performance liquid chromatography [HPLC], dry, QC: Mass Check) (available from Metabion, Planegg, Germany) and 5′-dabcyl-AGCTCCCAGGCTCAGATC-3′ (HPLC, dry, QC: Mass Check) at a final concentration of 0.25 µM. The reaction mixtures were incubated in a multilabel counter plate reader Victor 3 (Model 1420-051, Perkin Elmer, Waltham, MA, USA) for 10 min at 37 °C, followed by product quantification at 490/528 nm (excitation/emission wavelength). All experiments were performed in triplicate.

### 2.4. SARS-CoV-2 nsp14 Exonuclease Activity Assays 

Synthetic ssRNA substrate (RNA22, 5′-CGCAGUGAGCUCCUAAUCGCCC-3′) was commercially obtained from IDT [17]. Final reaction mixtures contained 20 mM HEPES (pH 7.5), 5 mM DTT, 10 mM Mg^2+^, 10 µM RNA22 substrate, and 0.5 µM of nsp14-nsp10 complex in the absence or presence of inhibitors. The reactions were incubated at 30 °C for 5 min. Reactions were stopped by heating at 95 °C for 5 min. Samples were centrifuged at 18,000× *g* for 15 min at 4 °C. Supernatants were collected and analyzed by HPLC. The amount of RNA22 substrate was analyzed on a 1260 Infinity II Agilent Technologies HPLC instrument equipped with a Dionex DNAPac PA-100 (Thermo Scientific, Rockford, IL, USA). The processed samples (10 µL) were injected onto a Dionex DNAPac PA-100 analytical column at a flow rate of 1 mL/min and eluted with a linear gradient of 10 to 2000 mM NH_4_HCO_3_ buffer (pH 7.8) for 90 min, followed by 30 min of equilibration to initial conditions. The HPLC column was maintained at 40 °C. Spectra were recorded at 256 nm. Open Lab CDS software was used to analyze and calculate the areas under the peaks in HPLC spectra. The area under the peak determined the amount of intact RNA22. IC_50_ was determined by plotting inhibitor concentration against the amount of remaining intact substrate. The background was corrected by removing the smallest response and data were normalized by dividing with the largest response. The data were analyzed and plotted using GraphPad Prism (9.4.0) software.

### 2.5. SARS-CoV-2 nsp14 N7-Methyltransferase Activity Assays 

A previously published protocol was modified to measure nsp14 MTase activity [51]. Assays were set up in a 25 µL reaction volume containing 40 mM Tris-HCl (pH 8.0), 5 mM DTT, 2 µM GpppA RNA cap analog (New England Biolabs, Ipswich, MA, USA), 10 µM adenosyl-methionine (AdoMet; Thermo Fisher, Rockford, IL, USA), and 1 µCi [3H] AdoMet (PerkinElmer, Waltham, MA, USA). Full-length SARS-CoV2 nsp14 was added at a concentration of 250 nM or as indicated in each reaction. DMSO (5% *v*/*v*) or test compound **1c** was added to each reaction sample. Nonradioactive AdoMet concentrations were adjusted to include 1 µCi [3H] AdoMet (PerkinElmer, Waltham, MA, USA) per reaction based on specific gravity provided by the supplier. ^7^MeGpppA RNA cap analog served as a negative control. Reaction mixtures were incubated at 37 °C for 1 h, and reactions were stopped by the addition of 25 µL of 1 mM adenosyl-homocysteine (AdoHcy; Thermo Fisher, Rockford, USA). Samples were prepared using filter binding assays. Samples were spotted on a DEAE-cellulose (3 MM, grade 81) paper (gifted by Dr. Lulu Xu, Cleveland Clinic, Cleveland, OH, USA) prewet with 40 mM Tris-HCl (pH 8.0) buffer. Samples were washed twice with 10 mM ammonium formate (Sigma-Aldrich, St. Louis, MO, USA) (pH 8.0), twice with MilliQ water, and once with absolute ethanol (Sigma-Aldrich, St. Louis, USA). Each wash lasted for 5 min with intermittent gentle rocking. After air drying for 15 min, the filter paper was cut and transferred to individual tubes containing beta plate scintillation fluid (PerkinElmer, Waltham, MA, USA). The amount of ^3^H label bound was measured in counts per minute using a Wallac scintillation counter. Graphs were plotted in Graph PAD prism. 

### 2.6. Drug Toxicity to Human Adenocarcinoma Lung Cells 

A549 cells (ATCC) were treated with varying concentration of **1c** ranging from 0.75 to 50 µM for 24, 48, 72, or 96 h in a 96-well plate. Percentages of live cells was determined and compared to DMSO (solvent) control using an Alamar-Blue-based fluorescence (Ex/Em 560/590 nm) assay as per the manufacturer’s protocol (ThermoFisher Scientific, Rockford, USA). The amount of fluorescence produced is proportional to the number of living cells. The experiments were carried out in triplicate.

### 2.7. SARS-CoV-2 Replication Inhibition in A549 ACE2^+^ Cells 

Approximately 80% confluent monolayer of A549 ACE2^+^ cells in 6-well plates were washed once with DMEM and infected with SARS-CoV-2 WA1/2020 strain at 0.05 MOI. The plates were placed on a rocker for 45 min at room temperature in compound-free conditions for virus adsorption. The solutions were then removed and replaced by DMEM containing **1c** at various concentrations (12.5, 25, 50 µM) and incubated at 37 °C in 5% CO_2_ for 24 or 48 h. As a control, the infected cells incubated in compounds-free medium were included throughout the experiment. The supernatants were harvested followed by titration for virus yield determinations by CPE assay in Vero E6 ACE2 cells. Vero E6 ACE2 cells were cultured in 96-well flat-bottom microplates at a density of 1.5 × 10^4^ cells per well for 24 h at 37 °C in 5% CO_2_ atmosphere. The cells were washed once with serum-free DMEM and infected with 10-fold serially diluted virus samples harvested from A549 ACE2^+^ cells. The virus-infected plates were incubated at room temperature for 45 min for virus adsorption. The virus solutions were then replaced with DMEM and further incubated at 37 °C in 5% CO_2_ atmosphere for 72 h. The virus titers were determined by the uptake and subsequent extraction of neutral red dye. Briefly, cells were incubated with 0.033% neutral red dye for 3 h at 37 °C. Free dye was washed from the wells, and dye that was taken up by cells was quantified using a microplate reader with absorbance recorded at 540 nm. Absorbance values were expressed as percentages of uninfected control cells, and TCID_50_ (50% tissue culture infectious dose) was determined using Prism software (GraphPad, La Jolla, CA, USA). The experiments were performed twice in triplicate. 

### 2.8. Plaque Reduction Assay 

Plaque reduction assays were performed as described previously [52]. Briefly, confluent monolayers of Vero E6 ACE2 cells were cultured in a 6-well tissue culture plate and infected with approximately 200 pfu of SARS-CoV-2 WA1/2020 strain. After 45 min on a rocker, the viruses were removed and replaced by overlay medium (DMEM containing 1% low-melting agarose and test compounds at different concentrations). After incubating the cultures for 3 d, the monolayers were fixed with 4% (*v*/*v*) formaldehyde solution and the agarose plugs were removed and stained with 1% (*w*/*v*) crystal violet solution. The concentration required to deduce the EC_50_ was calculated by regression analysis of the dose–response curves generated from these data [53]. The experiments were performed three times. 

### 2.9. Molecular Docking 

The 2-dimensional structure of compound **1c** was optimized for 3D using discovery studio and the structure was minimized using CHARMM force field in Accelrys discovery studio pipeline as described earlier [54,55]. The energy-minimized structure was first used to perform a blind docking on the AutoDock Vina program followed by site-specific docking using Glide (Schrodinger), where grids were restricted in the 10 best scoring binding poses. The lowest energy binding pose were selected based on free energy of binding (G_binding_). The structures were analyzed and visualized in pymol and UCSF Chimera 1.8. 

## 3. Results

### 3.1. Chemistry

**Scheme 1:**
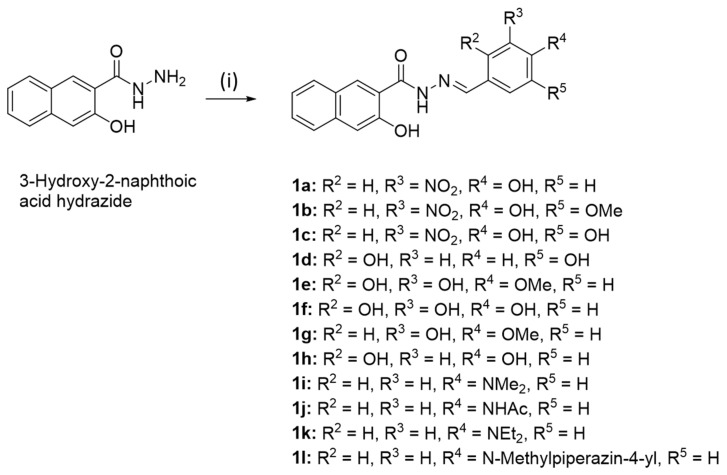
Reagents and conditions: (i) Aryl aldehyde, EtOH, reflux, 2 h, 49–87%.

The dynasore analogs **1a**–**l** were prepared from 3-hydroxy-2-naphthoic acid hydrazide and the corresponding aryl aldehydes in boiling ethanol (Scheme 1). Compounds **1a**–**c**, **1e,** and **1l** are new compounds, and their structures were confirmed by NMR, IR, and MS methods (see Supplementary Materials). Compounds **1d** and **1f**–**k** are known compounds, and analytical data were consistent with published data of these compounds [43,44,45,46,47].

**Scheme 2:**
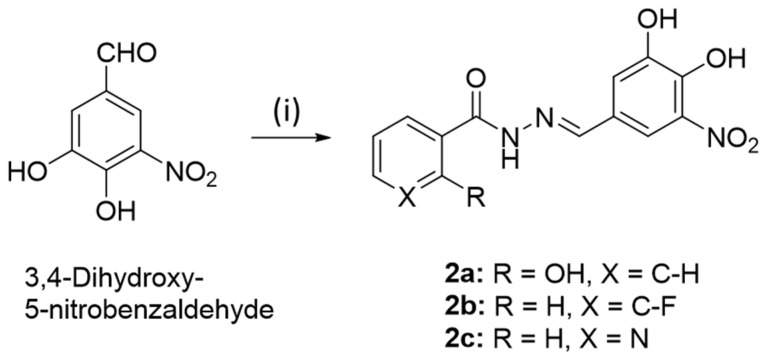
Reagents and conditions: (i) Acid hydrazide, EtOH, reflux, 2 h, 64–86%.

For reasons of comparison, the new compounds **2a**–**c** were prepared as close analogs of **1c** by reaction of 3,4-dihydroxy-5-nitrobenzaldehyde (obtained from 3-nitrovanillin refluxed in 48% HBr) and the corresponding acid hydrazide (Scheme 2). 

### 3.2. Inhibition of HIV-1 RNase H

Initially, dynasore and compounds **1a**–**l** and **2a**–**c** were evaluated for their inhibition of HIV-1 RNase H (Table 1). The HIV-1 RT-associated RNase H inhibition assay is well-established and suitable for the identification of hit compounds from compound series and libraries [49,50]. As expected, dynasore exhibited a considerable inhibition of HIV-1 RNase H, which was higher than the inhibition by the positive control RDS1643 [50]; however, addition of a nitro group to the dynasore structure in compound **1c** led to the strongest HIV-1 RNase H inhibitor of this series of test compounds, with an IC_50_ value of 0.57 µM. In terms of structure–activity relationships, it became clear that a catechol scaffold is necessary for a high HIV-1 RNase H inhibition, as evidenced by dynasore, **1c**, **1e,** and **1f**. The 2,3-dihydroxy-4-methoxyphenyl derivative **1e** and its 2,3,4-trihydroxyphenyl analog **1f** were slightly more active than the 3,4-dihydroxyphenyl parent compound dynasore, but less active than **1c**. Among the non-catechol derivatives, only 3-nitrovanillyl **1b** and 4-*N*-diethylaminophenyl **1k** revealed activities below 10 µM (IC_50_ = 7–8 µM). The latter compound was distinctly more active than its 4-*N*-dimethylamino analog **1i**, indicating an activity increasing effect by longer alkyl groups, while the corresponding acetamido and *N*-methylpiperazinyl derivatives **1j** and **1l** were inactive. Compounds **2a**–**c**, which are nitrocatechol analogs of **1c** differing in their acyl moiety, were much less active than **1c**. This underlines the importance of the 3-hydroxy-2-naphthoic acyl fragment in **1c** for HIV-1 RNase H inhibition.

### 3.3. Inhibition of ExoN Activity of SARS-CoV-2 nsp14/nsp10

**Figure 1:**
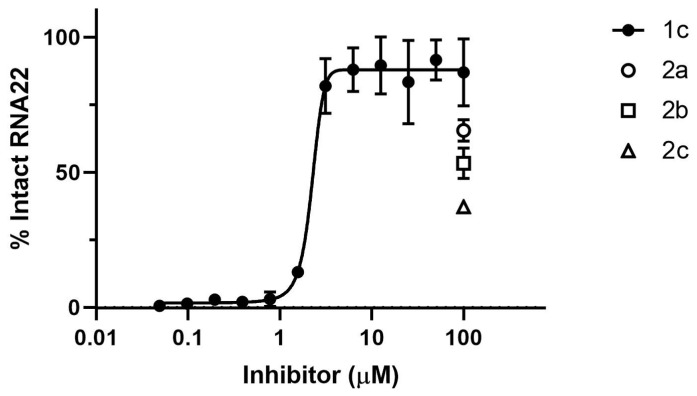
Inhibition of ExoN activity of nsp14/10 complex by **1c** and its analogs. ExoN activity of SARS-CoV-2 nsp14/nsp10 was determined at different concentrations of inhibitors in an in vitro assay using HPLC to monitor degradation of the RNA22 substrate. The inhibitors used were **1c** and its analogs **2a**, **2b**, and **2c**. An IC_50_ of 2.2 µM was calculated for inhibitor **1c**. Inhibition of ExoN activity by the analogs is shown at 100 µM concentration, as indicated on the graph. RNA22 substrate incubated under the same conditions (in the absence of both nsp14/10 and inhibitors) served as un-degraded control (100% intact substrate). Experiments were performed in triplicate. Error bars show standard deviations.

Based on its high HIV-1 RNase H inhibitory activity, compound **1c** was selected for SARS-CoV-2 ExoN exoribonuclease inhibition studies. Nsp10 interacts with the ExoN domain of nsp14 to stabilize it and stimulates its nuclease activity [23,56]. Degradation of the ssRNA substrate by nsp14/nsp10 complex was monitored using an HPLC-based assay in the absence or presence of the inhibitor. SARS-CoV-2 nsp14/nsp10 ExoN activity was strongly inhibited by **1c** with an IC_50_ value of 2.2 µM (Figure 1). Thus, the nsp14 inhibitory activity of **1c** lies in the range of the recently disclosed nsp14/nsp10 inhibitors patulin and ebselen [18,19]. The **1c** analogs **2a**, **2b**, and **2c**, were also tested to judge the importance of the 3-hydroxy-2-naphthoic acid scaffold for nsp14 inhibitory activity. But compounds **2a**–**c** had reduced inhibitory activities compared with **1c** (Figure 1). There was complete inhibition of the ExoN activity by **1c** at 100 µM, whereas **2a**, **2b,** and **2c** inhibited ExoN activity by about 65%, 53%, and 37%, respectively (Figure 1). 

### 3.4. Inhibitor **1c** Does Not Interfere with nsp14 MTase Activity

**Figure 2:**
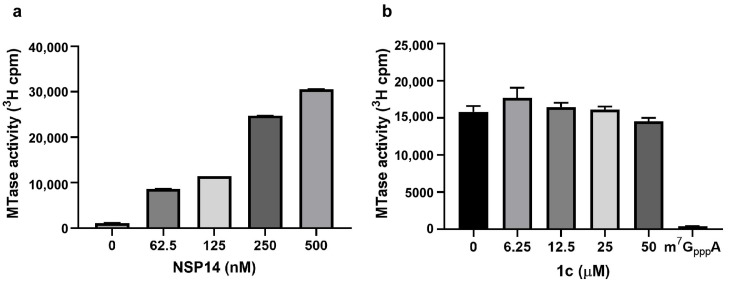
Effect of inhibitor **1c** on MTase activity of full-length SARS-CoV-2 nsp14. (**a**) Increasing concentrations of nsp14 were incubated with GpppA for 60 min in the presence of [3H]-SAM in a radioactive biochemical assay. (**b**) Nsp14 MTase activity monitored in the absence or presence of inhibitor **1c** using [3H]-SAM. m^7^GpppA served as a methylated control substrate, performed in the absence of **1c**. Experiments were performed in triplicate. Error bars show standard deviations.

Nsp14 is a bifunctional enzyme with ExoN (N-terminal) and N7-MTase (C-terminal) activities. The MTase activity is required for viral RNA capping, mRNA translation, and stabilization [51]. We tested the possibility of dual-function targeting by inhibitor **1c**. Full-length nsp14 MTase activity was assessed in a biochemical assay using synthetic GpppA mRNA cap substrate in the absence or presence of the inhibitor (Figure 2). Nsp14 methylates GpppA to m7GpppA by transferring the [3H]-CH_3_ moiety from [3H]-S-adenosyl methionine (SAM). Tritium incorporation in the methylated product was quantified using a DEAE-cellulose filter binding assay, followed by liquid scintillation counting. In positive control reactions, the recombinant purified nsp14 protein efficiently methylated the GpppA substrate in a concentration-dependent manner (Figure 2a). As negative controls, m^7^GpppA was not methylated, as determined by a background signal similar to incubations lacking nsp14. We observed no significant differences in the MTase activity as measured by tritium incorporation in the final product in the absence and presence of inhibitor **1c** (Figure 2b). Results indicate that **1c** did not interfere with the MTase activity of the nsp14 protein; therefore, it inhibits only its ExoN function. Moreover, inhibition of the nsp14 ExoN activity (due to **1c** binding) does not affect MTase domain function. 

### 3.5. Inhibitor **1c** Docks in the Catalytic Site of nsp14-nsp10 Complex

**Figure 3:**
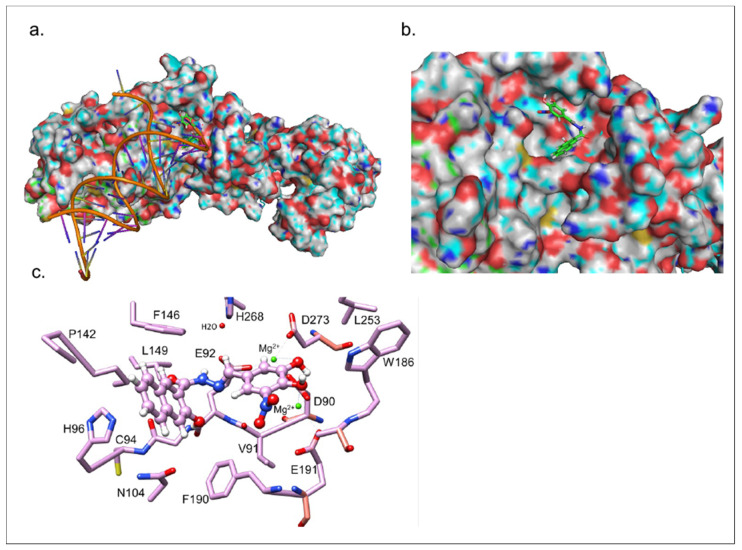
Here, **1c** binds in the exonuclease catalytic site of the nsp14-nsp10 complex. In silico docking simulation predicted specific binding of compound **1c** in the exonuclease catalytic site. (**a**) Electrostatic surface potential of nsp14-nsp10 complex with dsRNA substrate. (**b**) The binding pocket view of **1c** in the ExoN catalytic site. Negative (red) and positive (blue) surface potentials are shown. (**c**) Side chains of the nsp14 amino acid within 5 Å of compound **1c** in the ExoN catalytic site. Residues are depicted as sticks, while **1c** is depicted using ball-and-stick modeling with elemental color representations. Mg^2+^ ions are shown in green and the dotted line shows the possible interaction with Mg^2+^. Amino acid residues, as sticks, and inhibitor **1c**, in ball and sticks, are depicted using elemental colors.

The heterodimerization of nsp14 with nsp10 is a prerequisite for ExoN activity [56]. Therefore, to probe the molecular details of inhibitor **1c** interaction with nsp14, we utilized the high-resolution Cryo-EM structure of SARS-CoV-2 nsp10-nsp14 (WT)-RNA complex (PDB ID: 7N0B) for molecular docking simulation using Glide™ [57]. Initial docking simulations were performed using a large enough grid to include the entire complex. The best-scoring binding pose among the 10 top poses with RMSD of 2.5 Å preferentially occupies the catalytic site on the RNA-protein binding interface (Figure 3a–c). The in silico binding energy was calculated to be −7.2 kcal/mol. Inhibitor **1c** appears to bind to the EDD catalytic site residues, which are critical for substrate interaction [57]. The meta-hydroxyl group forms a hydrogen bond with aspartic acid 90, and glutamic acid 191 is in close proximity of the nitro group forming a salt bridge. 

### 3.6. Compound **1c** Does Not Affect Cell Viability

**Figure 4:**
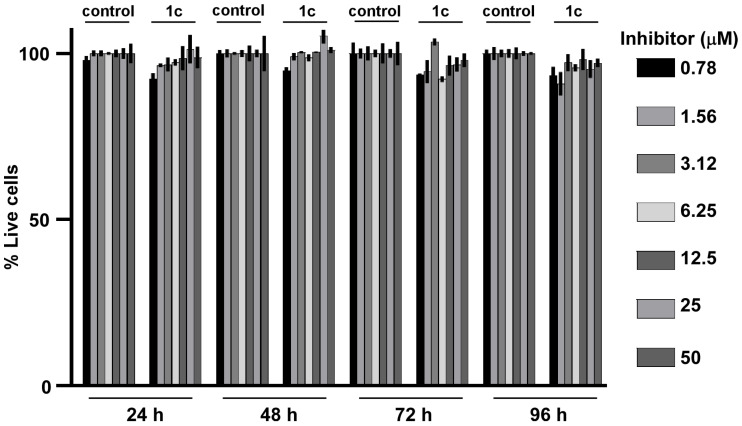
Compound **1c** was not toxic to A549 cells. A549 cells were treated with varying concentrations of **1c** (0.75 to 50 µM) for 24, 48, 72, or 96 h. Percent of live cells were determined and compared to DMSO (*v*/*v*) controls using an Alamar-Blue-based fluorescence (Ex/Em 560/590 nm) assay. The experiments were performed in triplicate. Error bars represent standard deviations.

To investigate whether **1c** might have drug-like properties suitable for controlling SARS-CoV-2 infections, its toxicity to human lung cancer cells was investigated. Only nontoxic compounds are suitable for antiviral drug development. Human A549 NSCLC (non-small-cell lung cancer) cells were applied for toxicity testing with **1c** (Figure 4). Compound **1c** showed no toxicity to A549 cells even at concentrations of 50 µM for four days. 

### 3.7. Compound **1c** Inhibits SARS-CoV-2 Replication

**Figure 5:**
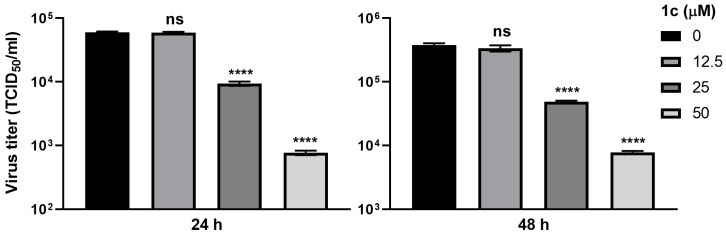
SARS-CoV-2 replication inhibition by **1c** in A549 ACE2^+^ cells. A549 ACE2^+^ cells were infected at an MOI of 0.05 and treated with varying concentrations of **1c** (12.5, 25, 50 µM) for 24 or 48 h. The experiments were performed twice in triplicate (N = 6). Statistical significance was calculated using unpaired *t*-test (ns, not significant; ****, *p* < 0.0001). Error bars represent standard deviations.

Next, the antiviral activity of compound **1c** was evaluated using A549 ACE2^+^ cells infected with SARS-CoV-2 (Figure 5). Treatment of infected cells at 12.5 µM of **1c** had no measurable effect in these assays. However, at 24 h post-infection, 25 µM of **1c** led to a 6.4-fold reduction in virus titers, while 50 µM of **1c** decreased viral titers by 78.5-fold. At 48 h post-infection, 25 µM of **1c** led to a 7.8-fold decrease in virus titers, while 50 µM of **1c** caused a 48.9-fold decrease. 

**Figure 6:**
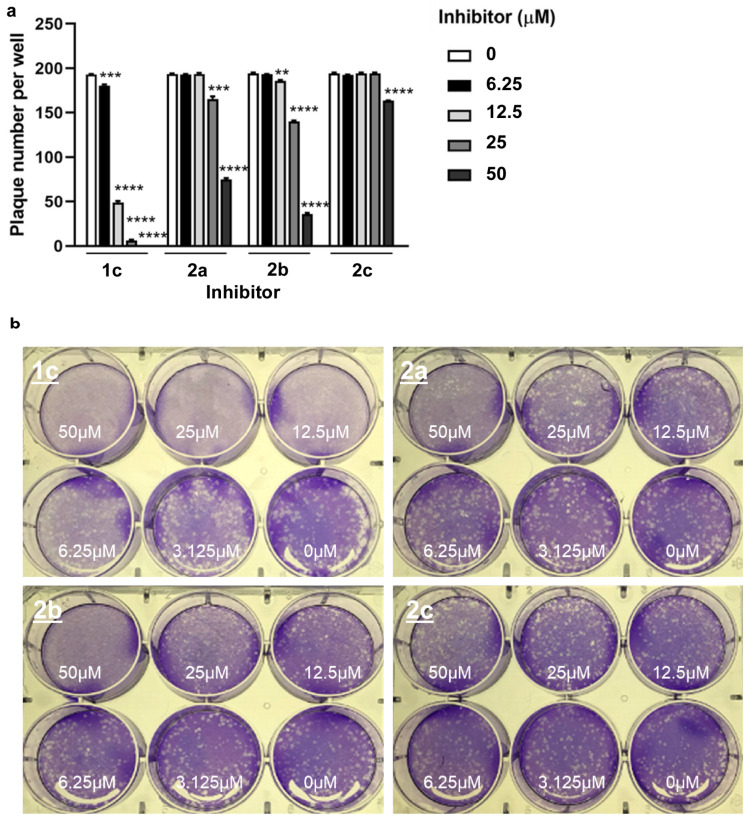
Plaque reduction assay. (**a**) SARS-CoV-2 replication inhibition by **1c** and **2a**–**c** at the indicated concentrations for 72 h. Statistical significance was calculated using unpaired *t*-test (**, *p* < 0.01; ***, *p* < 0.001; ****, *p* < 0.0001). (**b**) Representative images of wells in a plaque reduction assay are shown. Inhibitors and their concentrations are indicated on the panels.

To quantify the antiviral activities of **1c** and **2a**–**c** as EC_50_ values, plaque reduction assays were carried out (Figure 6, Table 2). The most active antiviral compound was **1c** with an EC_50_ value of 10.2 µM (Table 2). Analogs **2a** and **2b** showed weaker activity, while nicotinoyl derivative **2c** was inactive at concentrations of up to 50 µM (Table 2). EC_50_ of analogs **2a** and **2b** were 4.3- and 3.4-fold times higher compared to **1c**, respectively. The observed tendency of antiviral activity among compounds **1c** and **2a**–**c** is consistent with their nsp14/nsp10 ExoN inhibitory activity. These findings support inhibition of ExoN as the molecular mechanism for the antiviral activity of **1c**. About fivefold higher doses of **1c** were required for the inhibition of SARS-CoV-2 replication than for inhibition of ExoN nsp14/nsp10 activity in vitro, perhaps due to reduced cellular uptake of the drug. 

## 4. Discussion

In this study, the nontoxic dynasore analog **1c** was identified as a new dual HIV-1 RNase H and SARS-CoV-2 nsp14/nsp10 exoribonuclease inhibitor. In addition, **1c** blocked SARS-CoV-2 replication in infected cells; thus, it is a promising lead antiviral drug candidate suitable for further development. The DEDD motif ribonucleases HIV-1 RNase H and SARS-CoV-2 ExoN with Mg^2+^-coordinated active sites are both targets of **1c**. Further efforts towards the design of improved dual HIV-1 RNase H and SARS-CoV-2 nsp14/nsp10 inhibitors are currently underway. Notably, the non-nitro derivatives **1e** and **1f** with substituted catechol ring systems also displayed reasonable HIV-1 RNase H inhibitory activities, and can be considered for drug optimization studies to tackle SARS-CoV-2 infections more efficiently. Results obtained from MTase inhibition assays support the conclusion that ExoN and MTase domains are functionally independent, consistent with prior findings on SARS-CoV-2 and SARS-CoV nsp14 [22,51,58].

The natural product BTP (2,7-dihydroxy-4-isopropyl-cyclohepta-2,4,6,-triene or β-thujaplicinol, isolated from *Thuja plicata*) and various synthetic heterocyclic compounds were reported to possess similar nanomolar activity against HIV-1 RNase H [27,59,60]. Only a recently disclosed winged *N*-galloyl-*N*-sulfonylpiperazine-based HIV-1 RNase H inhibitor showed a higher activity [61]. In contrast, a 6-nitro-7,8-dihydroxycoumarin derivative exhibited distinctly lower HIV-1 RNase H inhibition than **1c** [62]. The nsp14 inhibitory activity of **1c** lies in the range of the recently disclosed nsp14/nsp10 inhibitors patulin and ebselen [18,19]. 

Because **1c** inhibits both HIV-1 RNase H and SARS-CoV-2 nsp14 ExoN, it is conceivable that **1c** might also inhibit other viral proteins with DEDD motif, such as nucleases of bunyaviruses (including Lassa virus, hantavirus, Rift Valley fever virus, and Crimean–Congo hemorrhagic fever virus), nidoviruses (in particular, all other coronaviruses), and hepatitis B virus (HBV RNase H) [26,63,64,65,66,67]. This might pave the way for new therapies for infections caused by these human pathogenic viruses.

Thus, **1c** adds to the current arsenal of clinically applied acyl-hydrazone-based drugs and anti-infectives [68,69]. Future synthetic efforts will deal with the replacement of the hydrazone core fragment by other molecular systems, which cannot undergo hydrolysis but conserve the activity of **1c**. In addition, prodrug strategies to mask the aromatic hydroxyl groups (e.g., by esterification) of **1c** might be considered if necessary.

The established ribonuclease inhibition suggests a vital role of the nitrocatechol moiety for the activity of **1c**; however, the 3-hydroxy-2-naphthoic component of **1c** also contributed significantly to viral nuclease inhibition. A conceivable interaction of the nitro-substituted catechol with the nuclease active site was corroborated by nsp14-nsp10 docking experiments. In addition, this scaffold is a crucial component of salient human catechol-*O*-methyltransferase (hCOMT) inhibitors such as entacapone, which are clinically applied for the treatment of Parkinson’s disease [70]. Available pharmacokinetic data of these hCOMT inhibitors are expected to be relevant and useful for the further development of **1c**, or a derivative of **1c**, as an antiviral drug.

## Figures and Tables

**Table 1:** Inhibitory concentrations ^1^ IC_50_ (μM) of dynasore and compounds **1a**–**l** and **2a**–**c** when tested against HIV-1 reverse transcriptase (RT)-associated RNase H activity. RDS1643 served as positive control.

Compound	HIV-1 RNase H	Compound	HIV-1 RNase H
**Dynasore**	3.10 ± 0.52	**1i**	27.54 ± 2.65
**1a**	69.56 ± 8.46	**1j**	>100 (91%) ^2^
**1b**	7.16 ± 2.35	**1k**	7.94 ± 2.17
**1c**	0.57 ± 0.04	**1l**	>100 (70%) ^2^
**1d**	35.95 ± 8.60	**2a**	13.99 ± 0.97
**1e**	1.02 ± 0.15	**2b**	15.23 ± 1.35
**1f**	0.93 ± 0.24	**2c**	24.68 ± 3.89
**1g**	>100 (84%) ^2^	**RDS1643**	10.8 ± 5.2
**1h**	36.38 ± 10.46		

^1^ Concentration required to reduce the HIV-1 RT-associated RNase H activity by 50%; values represent means of three independent experiments ± standard deviations. ^2^ Percentage of residual enzymatic activity at the highest concentration tested.

**Table 2:** EC_50_ values of **1c** and **2a**–**c** when tested for their anti-SARS-CoV-2 activity by plaque reduction assay.

Virus	EC_50_^1^ (μM)			
	1c	2a	2b	2c
SARS-CoV-2 WA1/2020	10.23 ± 0.01	43.97 ± 0.51	35.30 ± 0.59	>50

^1^ EC_50_ represents the concentration required to reduce the plaque number by 50%; values represent means and standard deviations of three independent experiments.

## Data Availability

Data are available from the authors upon reasonable request.

## Supplementary Materials

The following supporting information can be downloaded at: https://www.mdpi.com/article/10.3390/v15071539/s1, original ^1^H and ^13^C NMR spectra.
