# Supplementary material for: Analogs of the Catechol Derivative Dynasore Inhibit HIV-1 Ribonuclease H, SARS-CoV-2 nsp14 Exoribonuclease, and Virus Replication
Source: Viruses. 2023 Jul 13;15(7):1539. doi: 10.3390/v15071539 (PMC10385162; doi:10.3390/v15071539)

Supplementary Materials

Analogs of the Catechol Derivative Dynasore Inhibit HIV-1 Ribonuclease H, SARS-CoV-2 nsp14 Exoribonuclease, and Virus Replication

Abhishek Asthana [1], Angela Corona [2], Woo-Jin Shin [1], Mi-Jeong Kwak [1], Christina Gaughan [1], Enzo Tramontano [2], Jae U. Jung [1], Rainer Schobert [3], Babal Kant Jha [4], Robert H. Silverman [1,*] and Bernhard Biersack [3,*]

[1] Cancer Biology, Lerner Research Institute, Cleveland Clinic, 2111 East 96th St, Cleveland, OH 44106, USA; asthana@ccf.org, gaughac@ccf.org, shinw2@ccf.org, kwakm@ccf.org, jungj@ccf.org, silverr@ccf.org

[2] Laboratorio di Virologia Molecolare, Dipartimento di Scienze della Vita e Dell'Ambiente, Universitá degli Studi di Cagliari, Cittadella Universitaria di Monserrato SS554, 09042 Monserrato, Italy; angela.corona@unica.it, tramon@unica.it

[3] Organic Chemistry 1, University of Bayreuth, Universitätsstrasse 30, 95440 Bayreuth, Germany; rainer.schobert@uni-bayreuth.de, bernhard.biersack@yahoo.com

[4] Center for Immunotherapy and Precision Immuno-Oncology, Lerner Research Institute and Department of Translational Hematology and Oncology Research, Taussig Cancer Institute, Cleveland Clinic, 2111 East 96th St, Cleveland, OH 44195, USA; jhab@ccf.org

* Correspondence: silverr@ccf.org (R.H.S.), bernhard.biersack@yahoo.com (B.B.)

^{1}H NMR spectrum of **1a**

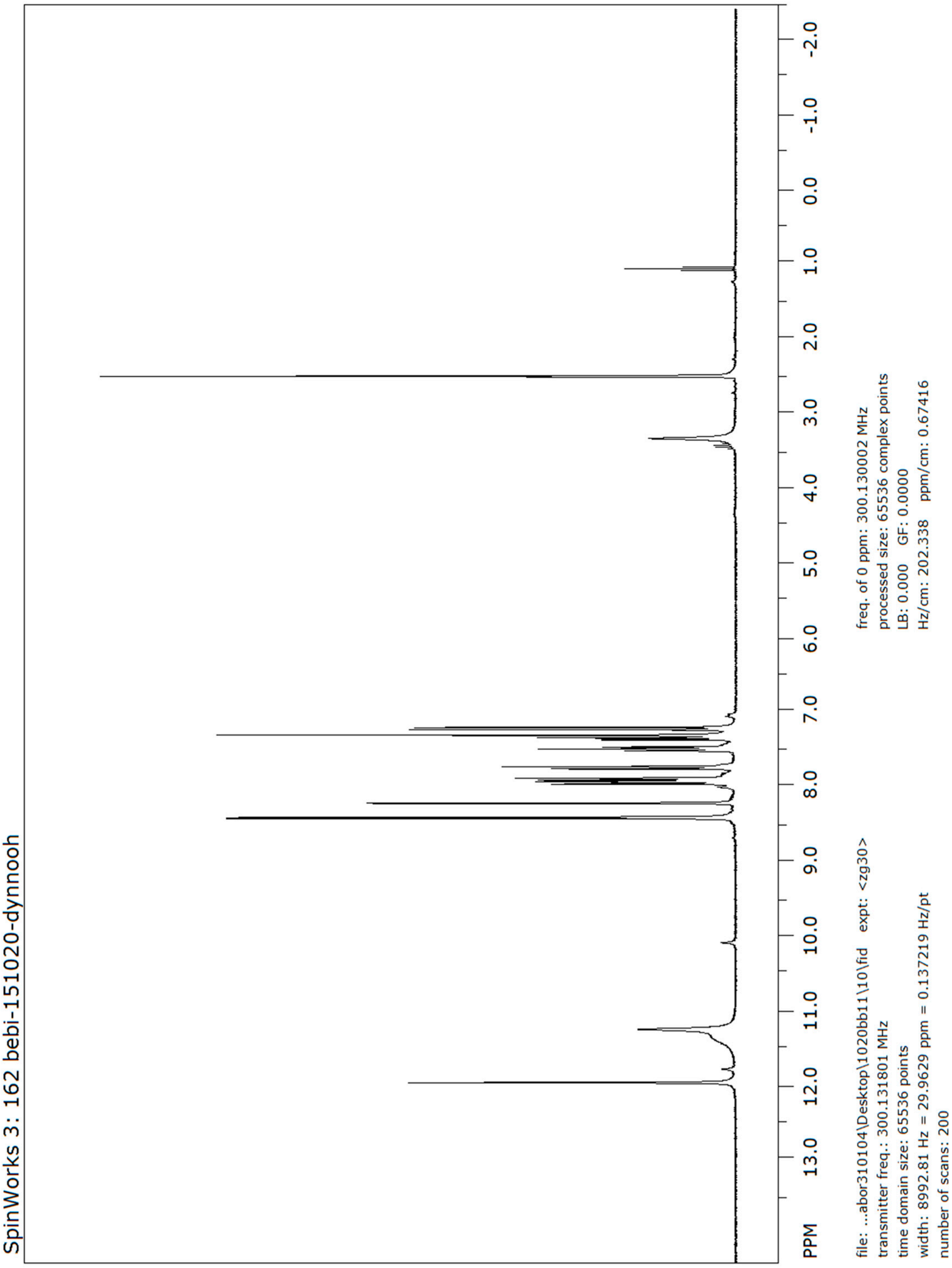

^{13}C NMR spectrum of **1a**

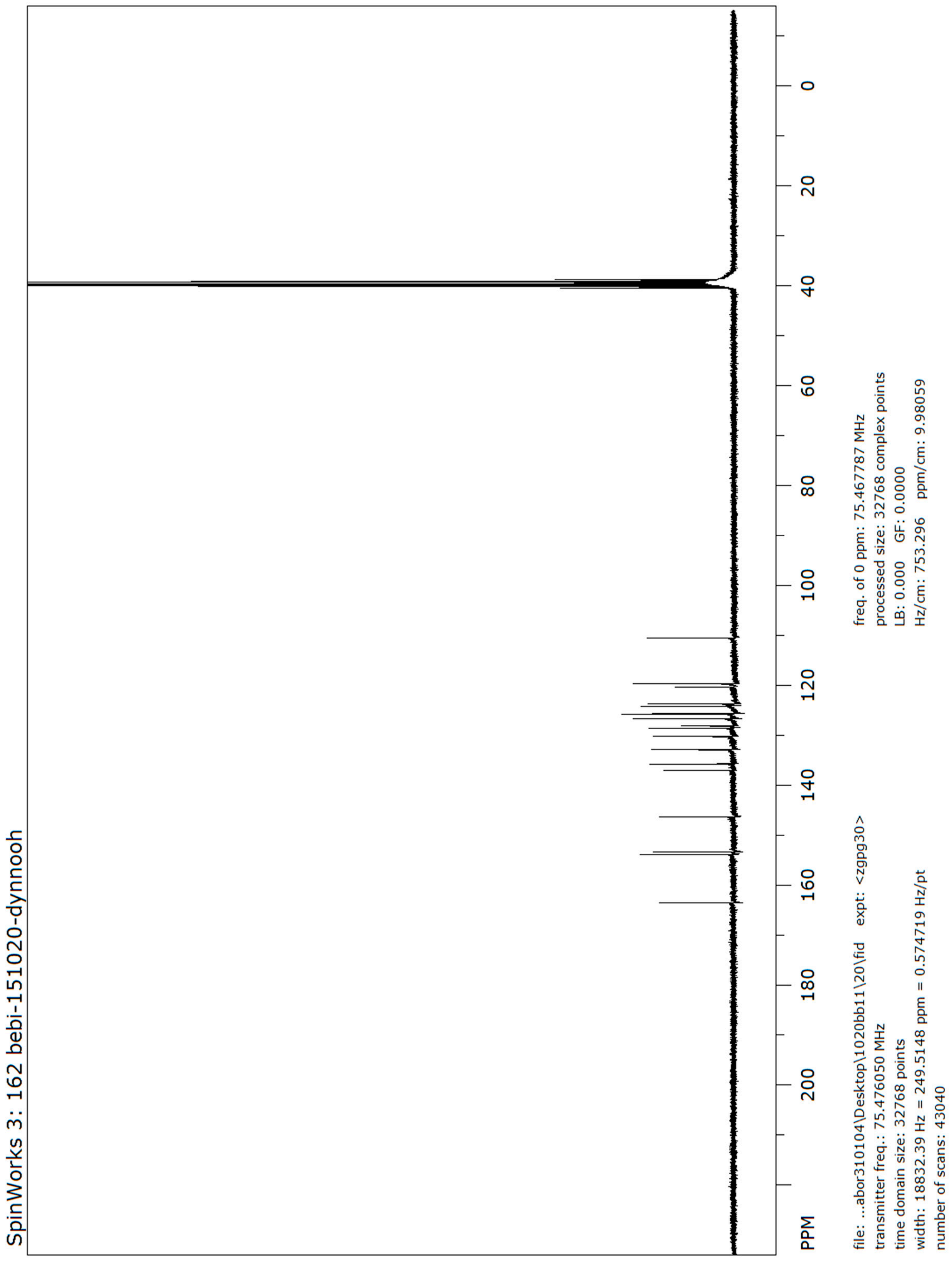

^{1}H NMR spectrum of **1b**

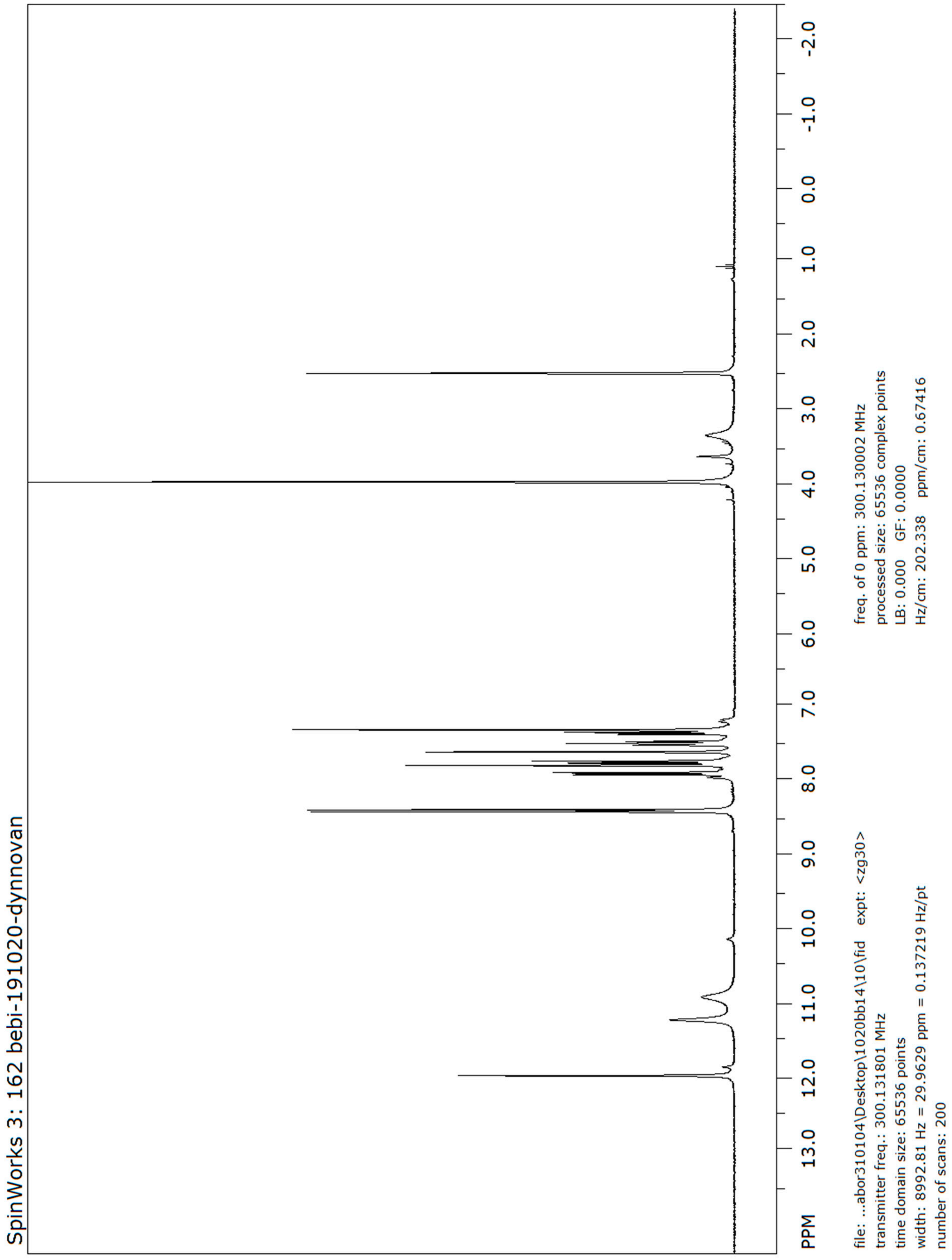

^{13}C NMR spectrum of **1b**

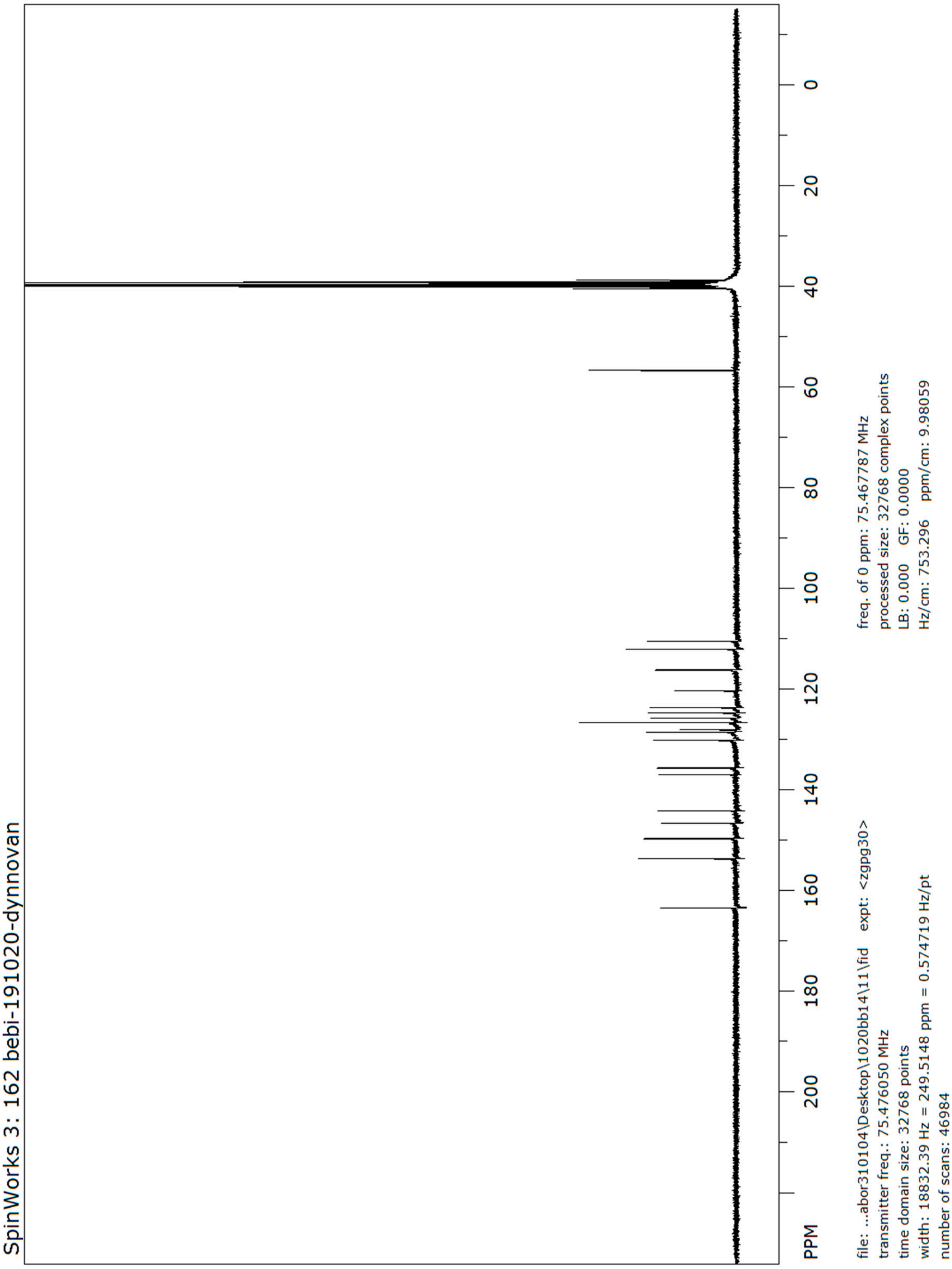

^{1}H NMR spectrum of **1c**

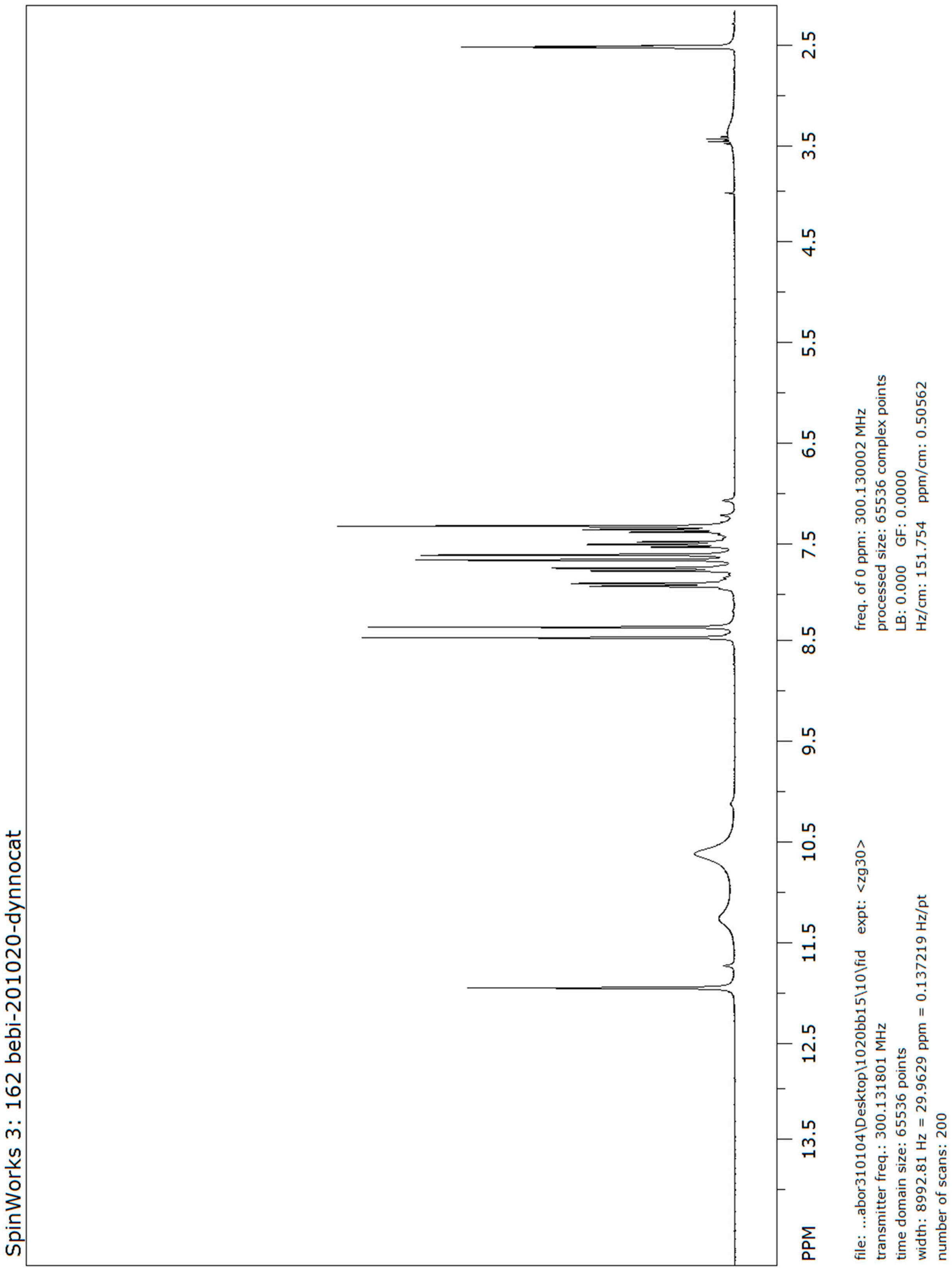

^{1}H NMR spectrum of **1c** (landscape format)

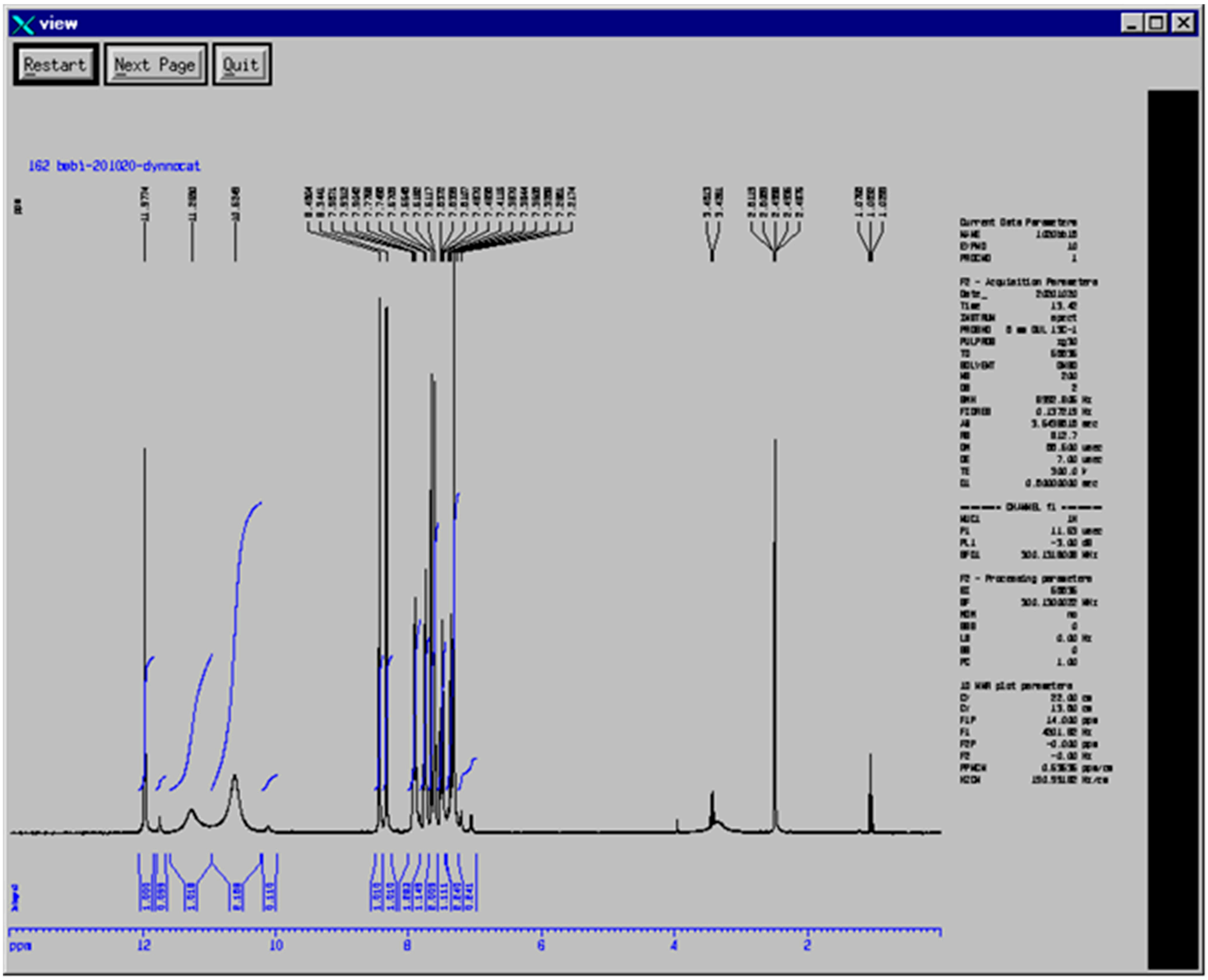

^{13}C NMR spectrum of **1c**

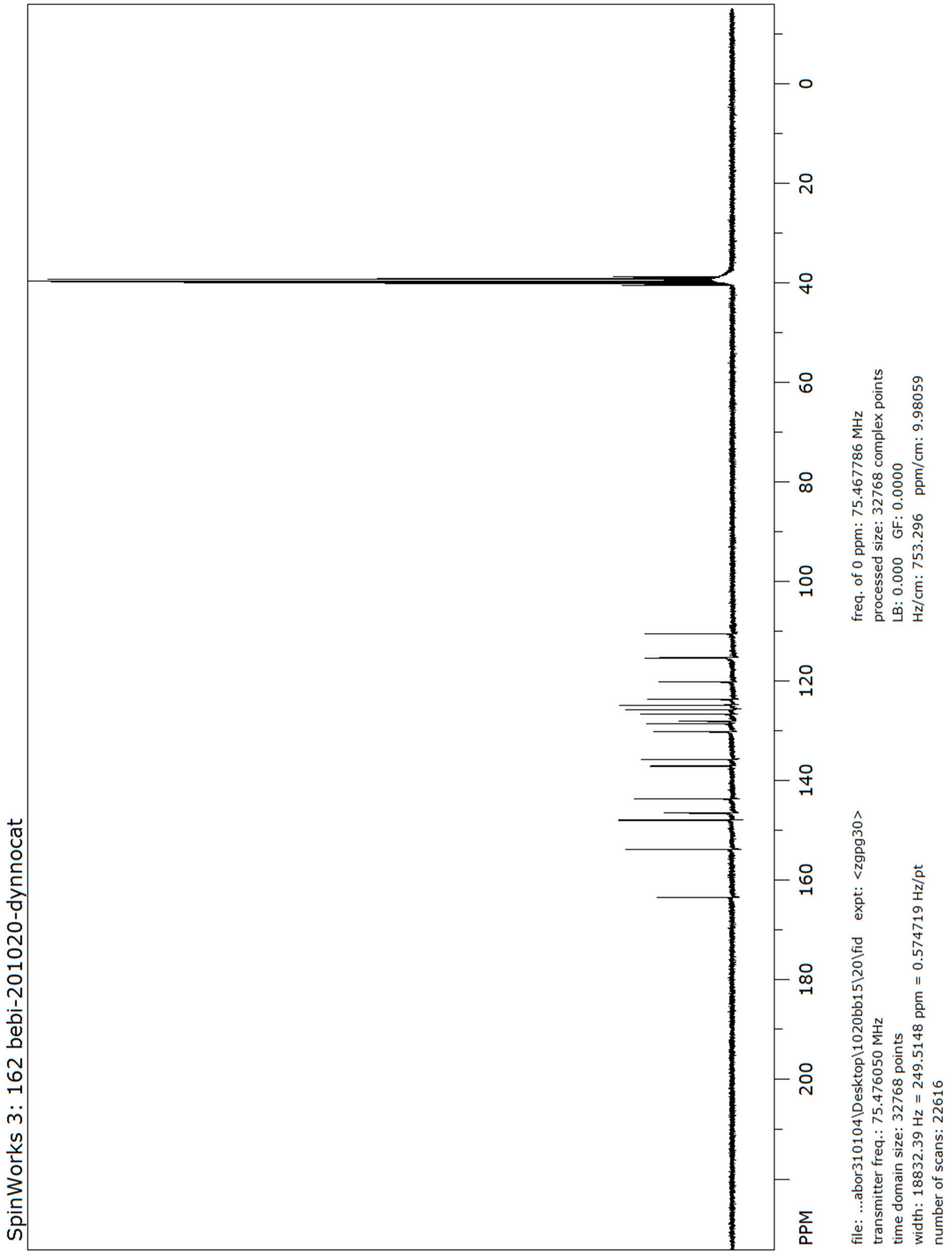

^{1}H NMR spectrum of **1e**

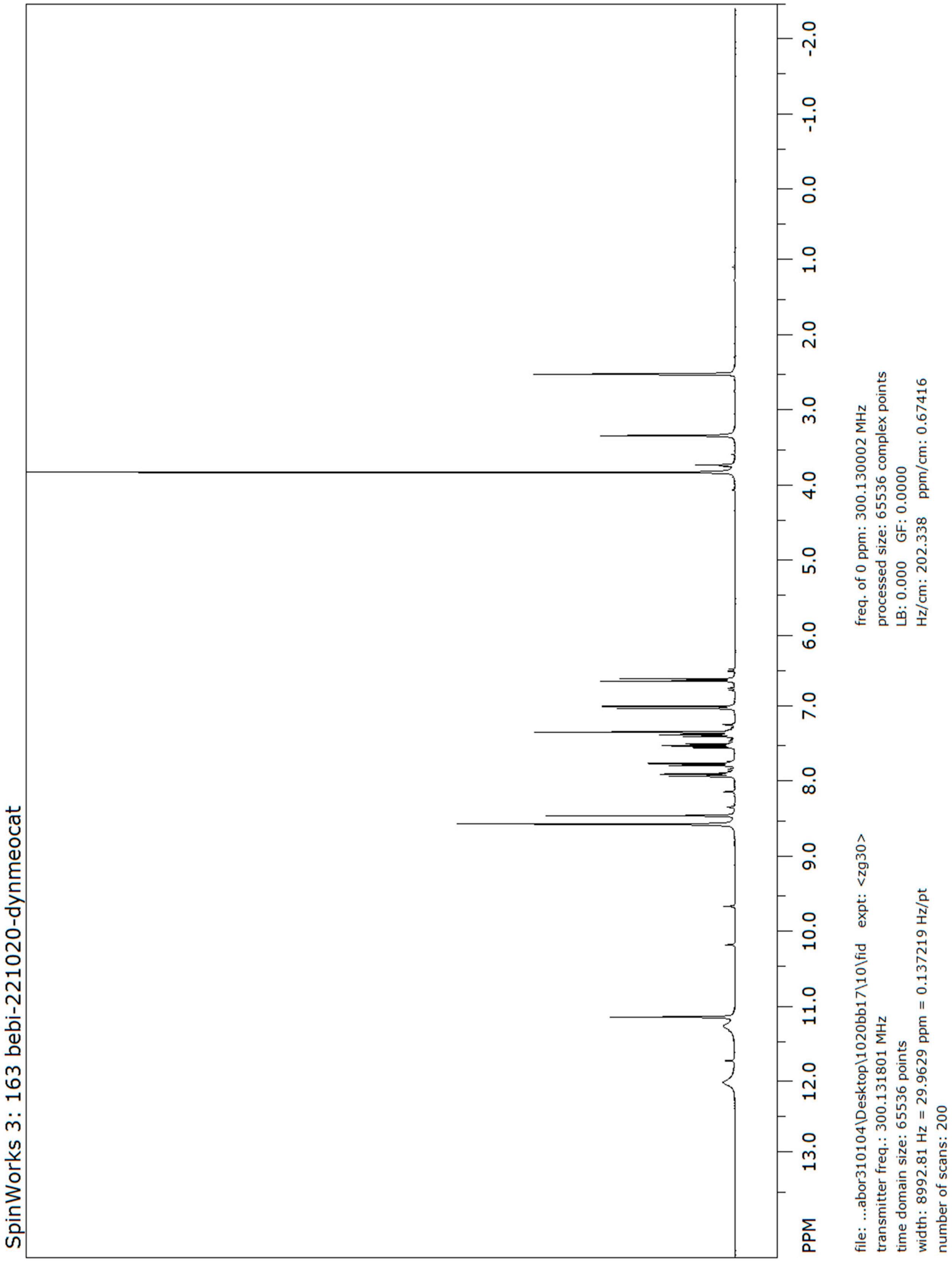

^{13}C NMR spectrum of **1e**

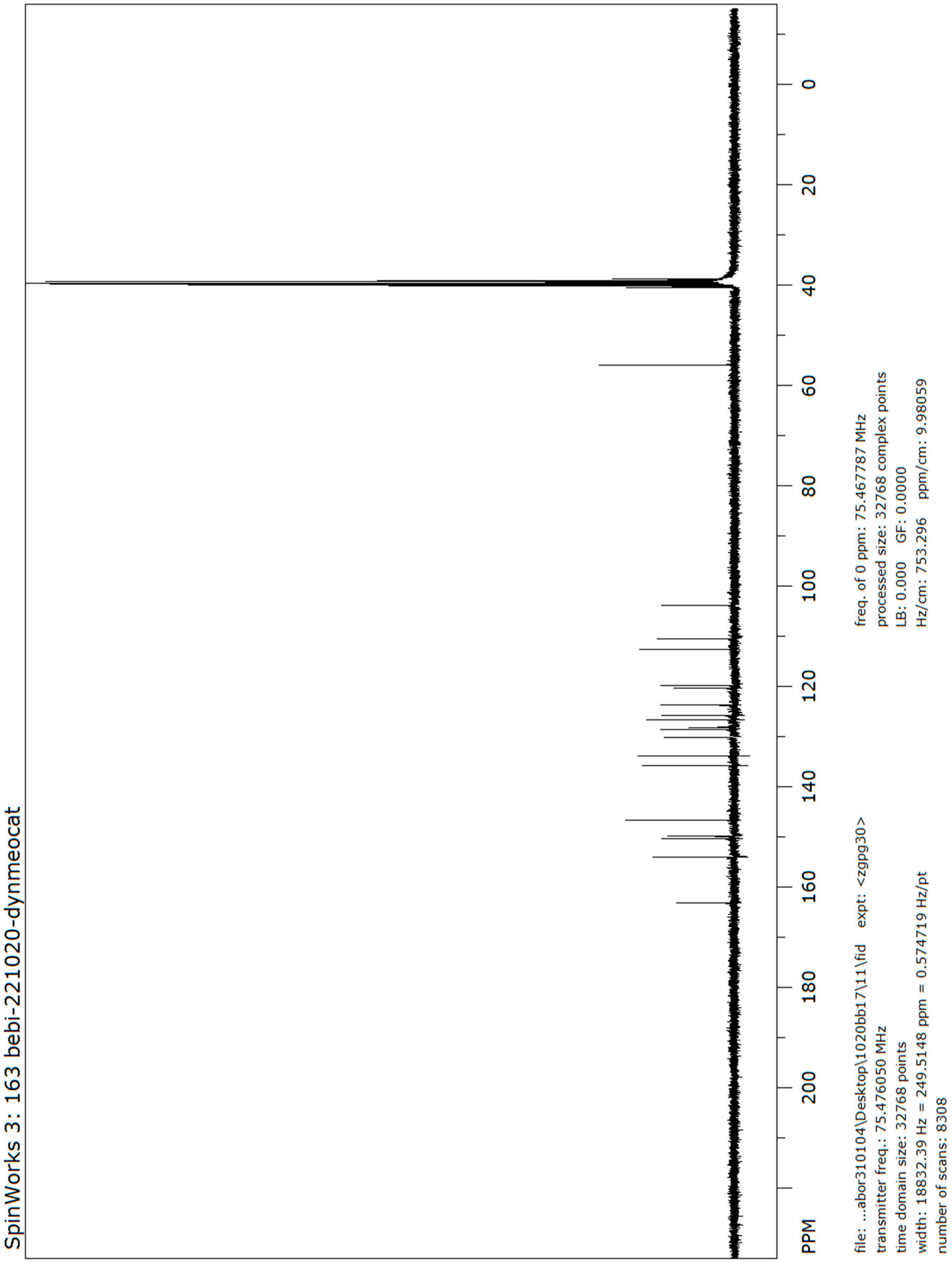

^{1}H NMR spectrum of **1l**

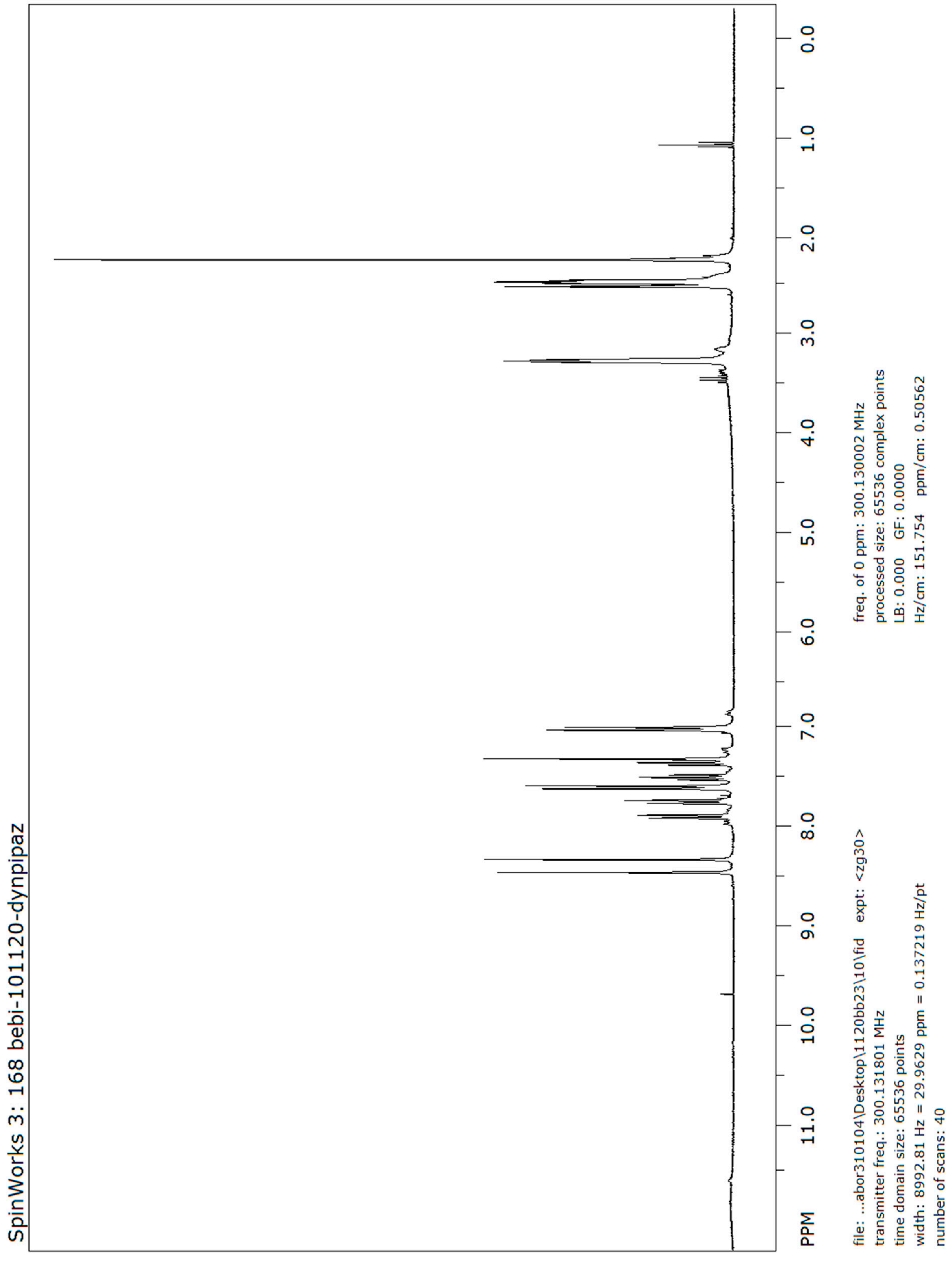

^{13}C NMR spectrum of **1l**

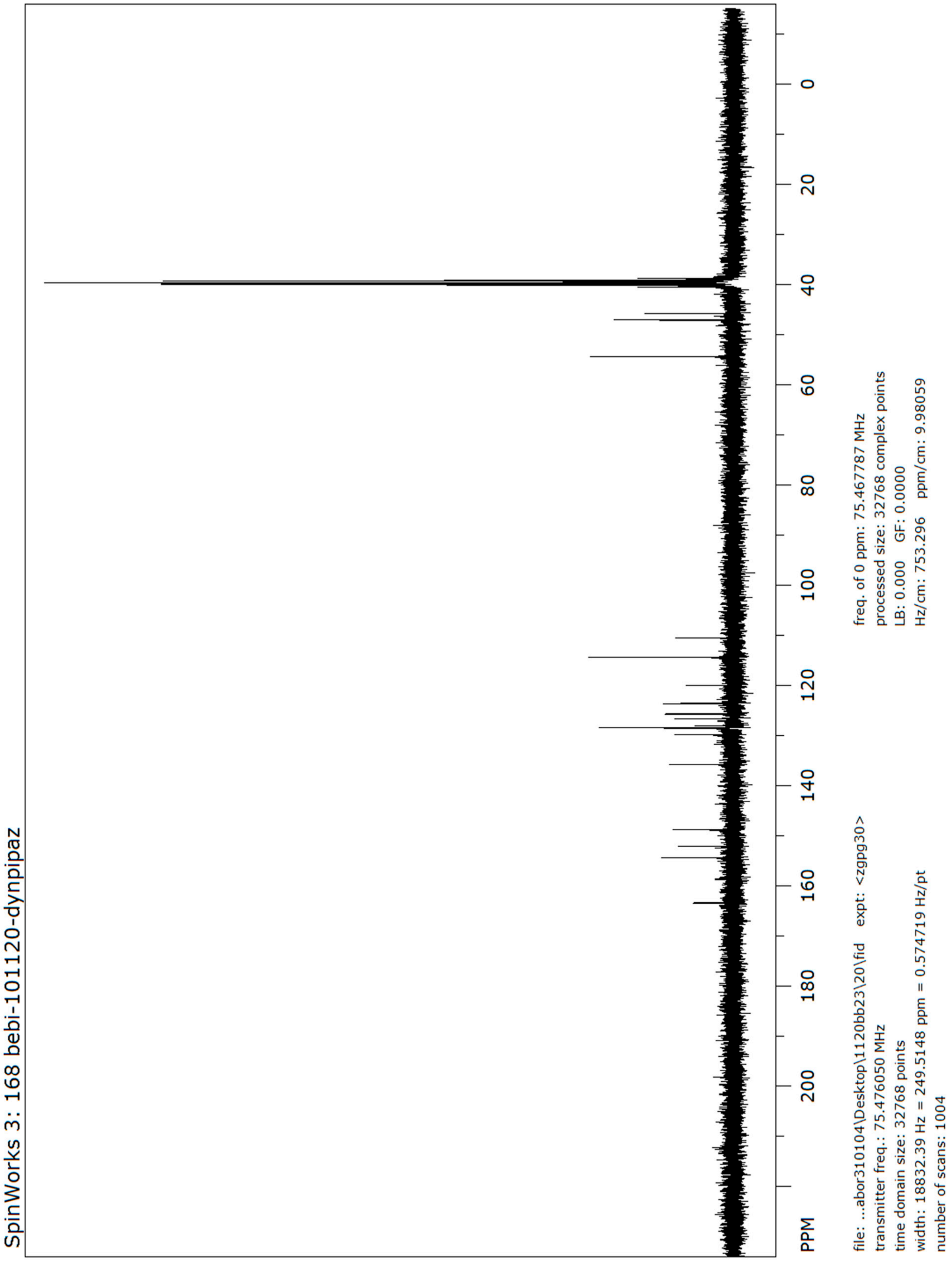

^{1}H NMR spectrum of **2a**

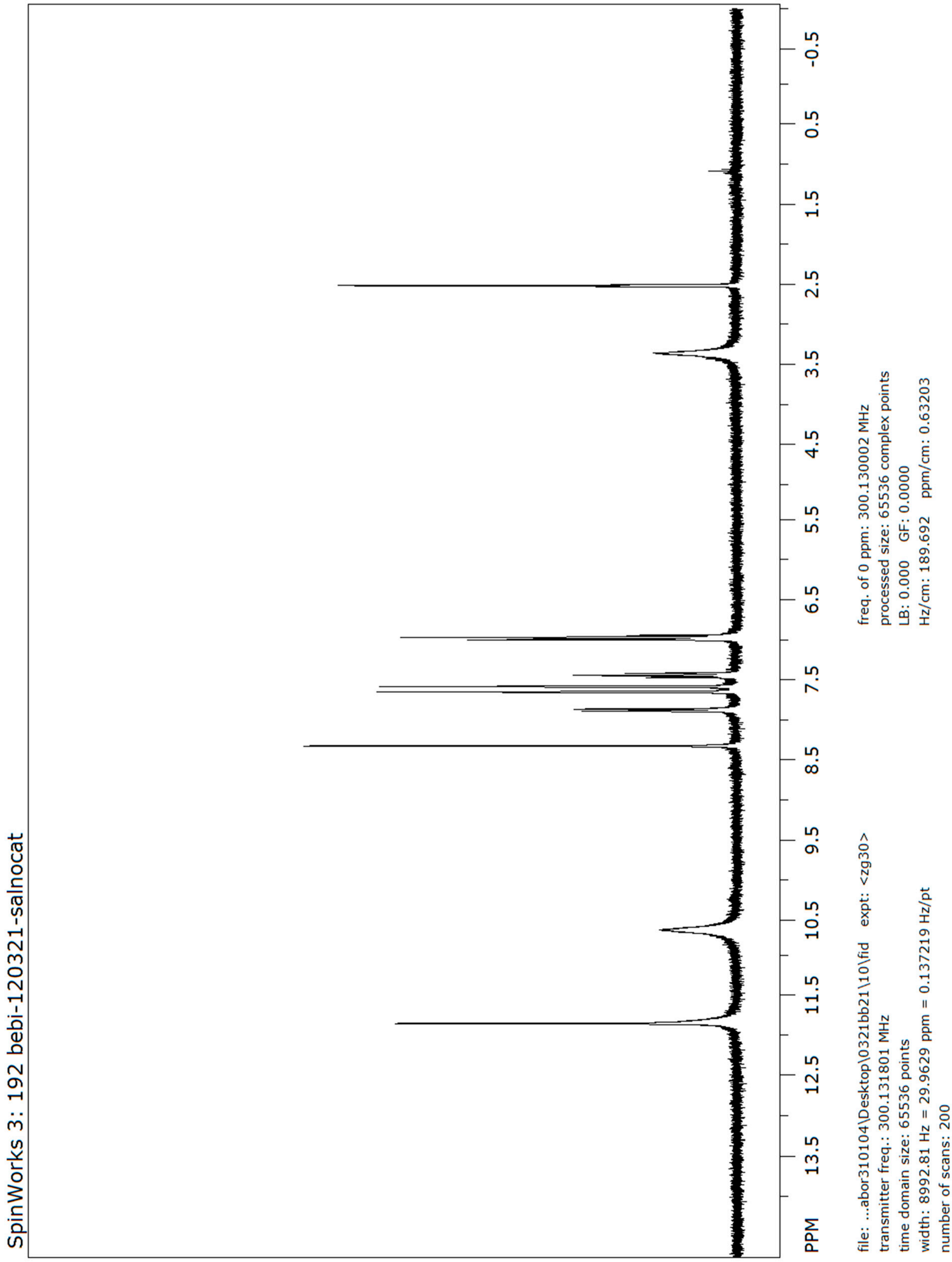

^{1}H NMR spectrum of **2b**

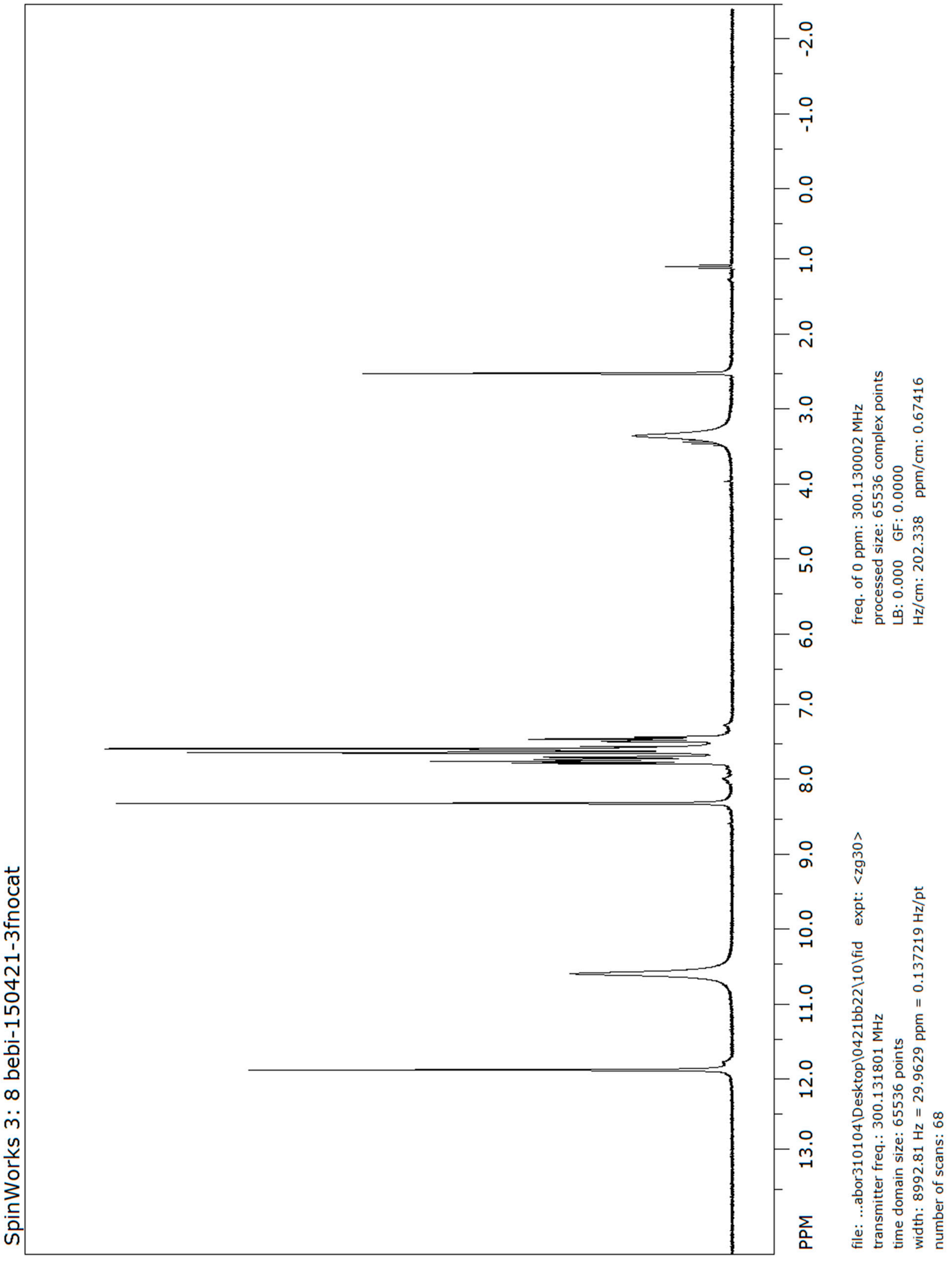

^{13}C NMR spectrum of **2b**

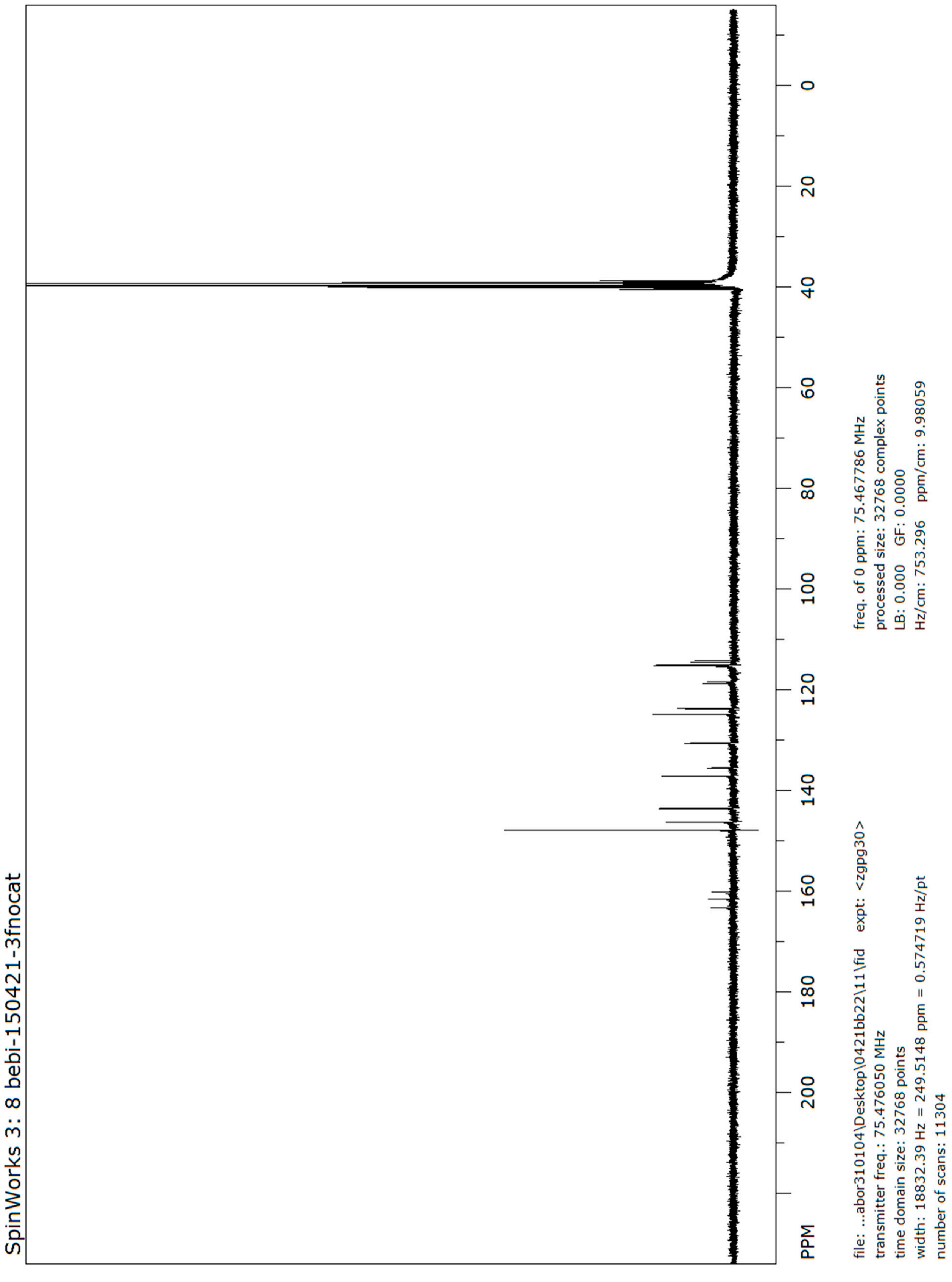

^{1}H NMR spectrum of **2c**

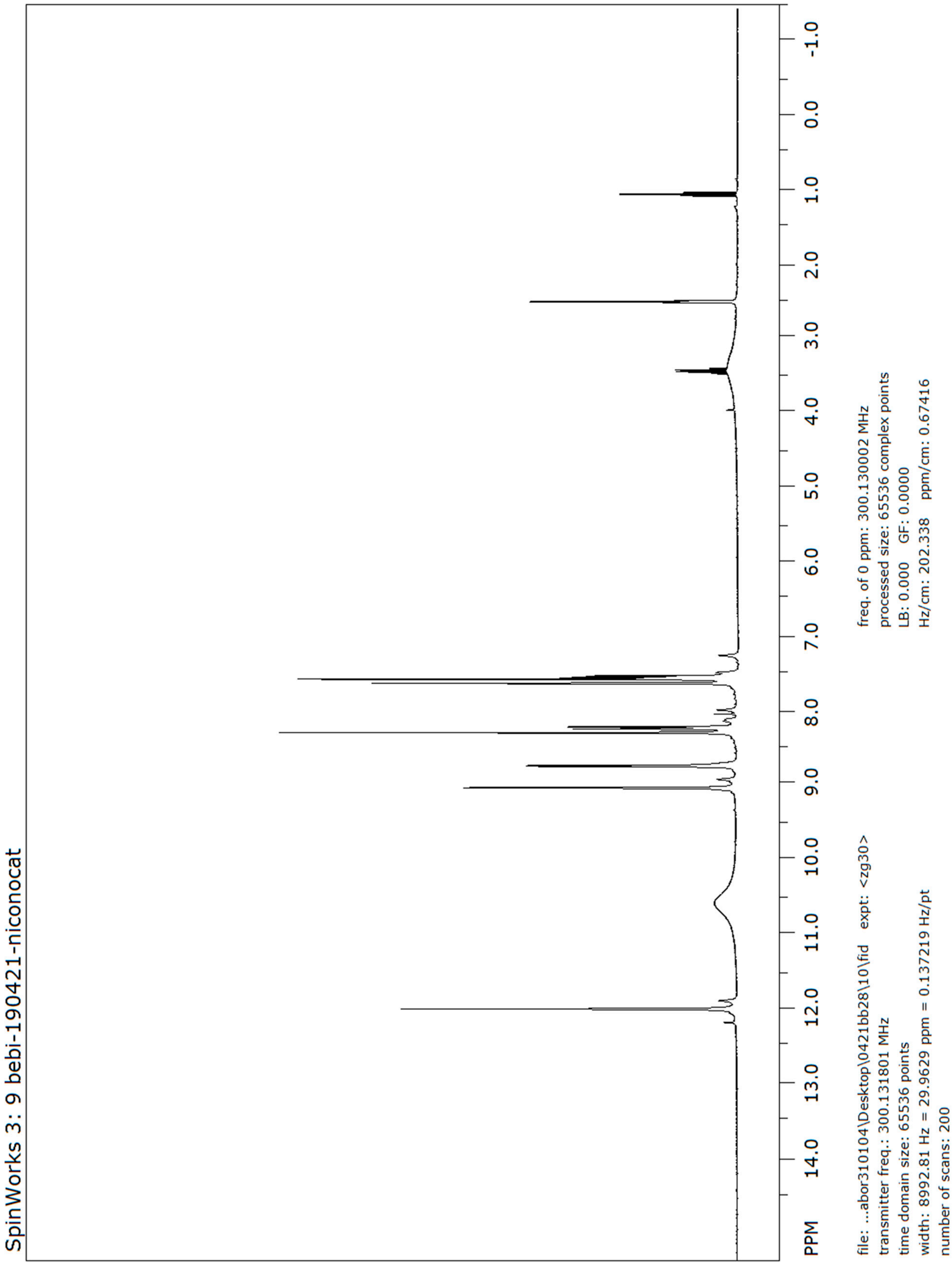

^{13}C NMR spectrum of **2c**

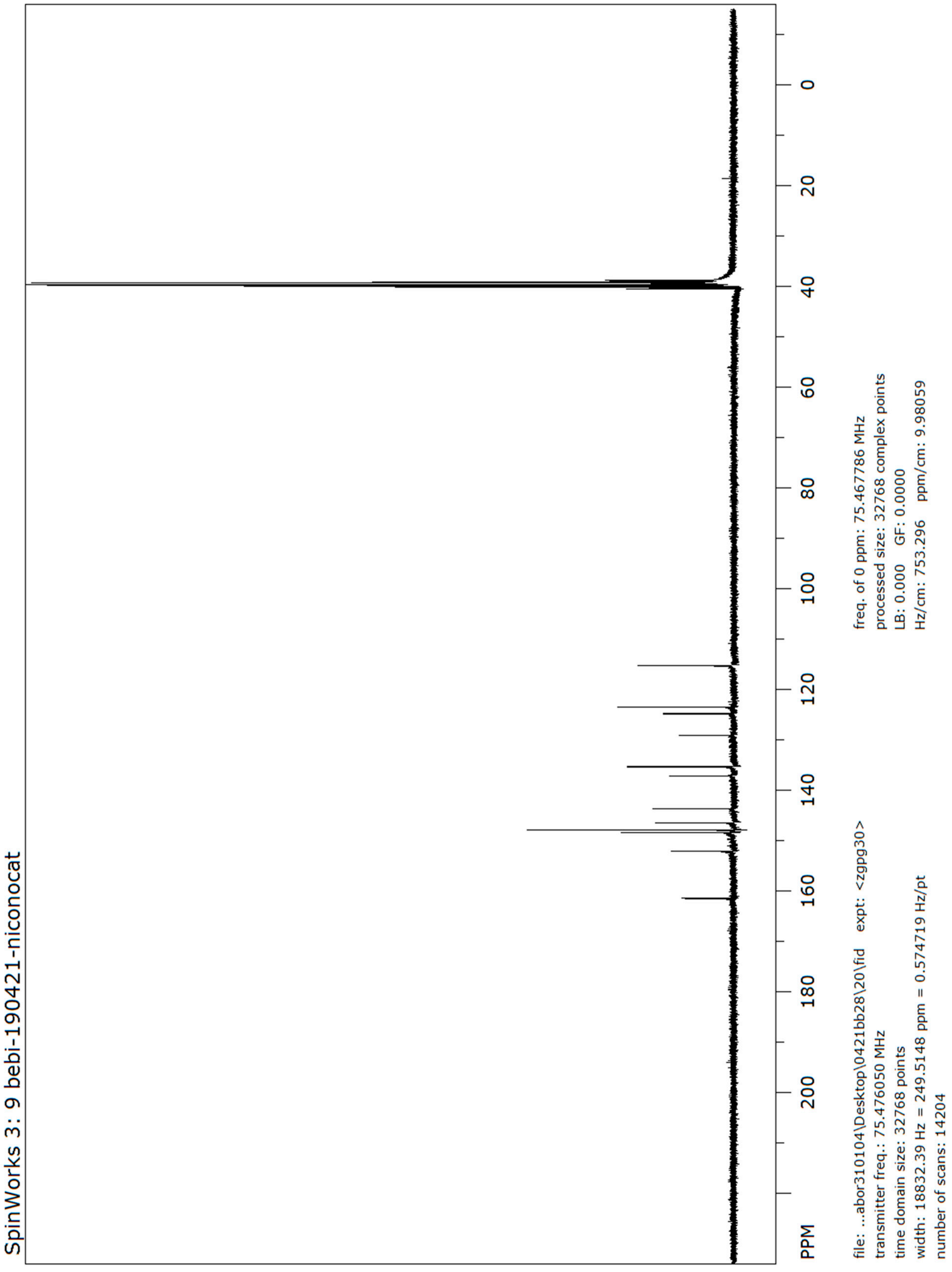

Supplement: Supplementary file 1 [file viruses-15-01539-s001.zip › viruses-2494252-supplementary.pdf]
